# Adipose-specific deletion of the cation channel TRPM7 inhibits TAK1 kinase-dependent inflammation and obesity in male mice

**DOI:** 10.1038/s41467-023-36154-3

**Published:** 2023-01-30

**Authors:** Weiting Zhong, Mingming Ma, Jingwen Xie, Chengcui Huang, Xiaoyan Li, Min Gao

**Affiliations:** 1 Department of Pharmacy, the Sixth Affiliated Hospital, Sun Yat-sen University, 510655 Guangzhou, China; 2 Department of Pharmacology, Cardiac and Cerebral Vascular Research Center, Zhongshan School of Medicine, Sun Yat-sen University, 510080 Guangzhou, China

**Keywords:** Endocrine system and metabolic diseases, Obesity

## Abstract

Chronic inflammation of white adipose tissue is a key link between obesity and the associated metabolic syndrome. Transient receptor potential melastatin-like 7 (TRPM7) is known to be related to inflammation; however, the role of TRPM7 in adipocyte phenotype and function in obesity remains unclear. Here, we observe that the activation of adipocyte TRPM7 plays an essential role in pro-inflammatory responses. Adult male mice are used in our experiments. Adipocyte-specific deficiency in TRPM7 attenuates the pro-inflammatory phenotype, improves glucose homeostasis, and suppresses weight gain in mice fed a high-fat diet. Mechanistically, the pro-inflammatory effect of TRPM7 is dependent on Ca^2+^ signaling. Ca^2+^ influx initiated by TRPM7 enhances transforming growth factor-β activated kinase 1 activation via the co-regulation of calcium/calmodulin-dependent protein kinase II and tumor necrosis factor receptor-associated factor 6, leading to exacerbated nuclear factor kappa B signaling. Additionally, obese mice treated with TRPM7 inhibitor are protected against obesity and insulin resistance. Our results demonstrate TRPM7 as a factor in the development of adipose inflammation that regulates insulin sensitivity in obesity.

## Abstract (web summary)

Adipose tissue inflammation contributes to the pathogenesis of obesity-related metabolic disorders. Here the authors report that adipocyte-specific genetic deficiency or pharmacological inhibition of TRPM7, a cation channel, attenuates adipose tissue inflammation, improves glucose homeostasis, and suppresses weight gain in obese male mice.

## Introduction

Obesity is a global epidemic that results from caloric oversupply and a positive energy balance^1^. It leads to multiple metabolic complications and comorbidities, such as type 2 diabetes mellitus, cardiovascular diseases, nonalcoholic fatty liver disease, and neurodegenerative diseases^1,2^. Obesity modulates the adipose tissue immune cell profile from anti-inflammatory to pro-inflammatory states, and an excessive immune response in white adipose tissue (WAT) may lead to a state of chronic, low-grade inflammation in case of nutritional changes^3^. In response to overnutrition, pro-inflammatory lipid species, cytokines and chemokines released by adipocytes, such as free fatty acids, TNF-α, MCP-1, Ccl5, etc., stimulate the recruitment of immune cells, especially pro-inflammatory macrophages, to fat pads and induce insulin resistance and diabetes^3^. Although several mechanisms of adipose inflammation induction have been proposed^3,4^, the causal relationship between obesity, and WAT inflammation, and the initial inflammatory trigger in adipose tissue is unclear.

Calcium signaling is considered as a vital event in the regulation of adipocyte function. Intracellular Ca^2+^ homeostasis in adipocytes is required for insulin transduction, lipid storage, and adipogenesis^5^. In elderly people with obesity, basal [Ca^2+^]_i_ levels are elevated in isolated adipocytes, and calcium channel blockers reportedly prevent the development of insulin resistance, restore adipocyte glucose uptake, and reduce diabetes mortality^6–8^. Adipocyte cytosolic Ca^2+^ induces nuclear factor of activated T-cell (NFAT) transcription factors^9^, which promotes the production of inflammatory cytokines in immune cells, and the ablation of NFATc2 and NFATc4 has been shown to prevent obesity and glucose imbalance in mice^10^. Furthermore, the key inflammatory kinase c-Jun NH_2_-terminal kinase (JNK) was reportedly activated in the adipose tissue of individuals and animal models with obesity^5^, which corresponds with the findings of our previous study showing that Ca^2+^ influx significantly blocks insulin action via calcium/calmodulin-dependent protein kinase II (CaMKII)-JNK activation in the adipocytes of hypertensive mice^11^. These findings support the essential role of Ca^2+^ fluxes in the activation of inflammatory signaling pathways. Understanding the molecular basis of Ca^2+^-permeable channels is, therefore, essential to elucidate adipocyte function in WAT inflammation.

Transient receptor potential (TRP) channels constitute a large family of cation-permeable channels, that are widely expressed in both non-excitable and excitable cell types^12^. However, the function of TRP channels in adipocytes remains largely undefined. TRP melastatin-like 7 (TRPM7), a member of TRPM subgroup of TRP channels, is a ubiquitously expressed nonselective cationic ion channel with its C-terminal serine-threonine kinase often referred to as a chanzyme^13^. TRPM7 is permeable to Ca^2+^, Mg^2+^, Zn^2+^, and Na^+^, and TRPM7 channel-mediated Ca^2+^ signals have been implicated in numerous physiological and pathological processes, such as phenotypic switching of vascular smooth muscle cells^14^, actomyosin contractility^15^, atrial fibrillation^16^, and egg activation during fertilization^17^. Moreover, TRPM7 channel participates in obesity-associated disorders. Shin et al^18,19^. reported that the TRPM7 blocker FTY720 eliminates obesity-induced hypertension by inhibiting channel activity. In addition, TRPM7 has emerged as an inflammation regulator with both positive and negative effects. The TRPM7 channel is sensitive to multiple signals pertinent to inflammation, such as extracellular pH^20^ and caspase-mediated cleavage^21^. In rheumatoid arthritis, suppression of TRPM7 activity causes endoplasmic reticulum stress to induce fibroblast-like synoviocyte apoptosis^22^. Further, TRPM7 is indispensable for T cell development^23^ and Fas-receptor-induced T cell apoptosis^21^. TRPM7 inhibition hinders TNF-α production and macrophage polarization^24^, and Ca^2+^ influx initiated by TRPM7 is necessary for toll-like receptor 4 (TLR4) receptor endocytosis and nuclear translocation of nuclear factor kappa B (NF-κB) during macrophage activation^25^. In addition, TRPM7 channel activation facilitates IL18-induced vascular calcification under pro-inflammatory conditions^26^ and contributes to platelet activity and thromboinflammation^27^. In contrast, reduced TRPM7 function leads to cardiac, vascular, and renal inflammation attributed to abnormal macrophage activation^28^. The paradox of TRPM7-regulated inflammation may be due to the distribution of TRPM7 in diverse cell types and differences in cellular function. However, the pathophysiological role of TRPM7 in obesity-induced adipose inflammation remains unclear.

Here, we report the pro-inflammatory role of TRPM7 in adipocytes under obese condition. Following the observation that the TRPM7 protein expression levels and channel activity were upregulated in adipose tissue and mature adipocytes of high-fat diet (HFD)-fed mice, we generated an adipocyte-specific TRPM7-knockout (TRPM7 ATKO) mouse model. The enhanced expression of TRPM7 in adipocytes promotes adipose tissue inflammation, resulting in glucose intolerant and insulin resistance. Adipocyte-specific elimination of TRPM7 maintains insulin sensitivity and protects mice from diet-induced obesity and adipose inflammation. This was explained by the reduced activity of the transforming growth factor-β activated kinase 1 (TAK1)-NF-κB cascade owing to the TRPM7-dependent-Ca^2+^ signal. We demonstrated that Ca^2+^ elevation through the TRPM7 channel substantially evoked TAK1 activation. We observed that cooperation of CaMKII and TRAF6 is required for TRPM7-mediated TAK1 activation, indicating that TAK1 is essential for the linkage between TRPM7-dependent-Ca^2+^ signal and pro-inflammatory signaling. Further, oral administration of TRPM7 inhibitor FTY720 on obese mice protected against obesity and insulin resistance. These findings revealed an unexpected role of TRPM7 in the inflammatory pathways in adipocytes that modulate obesity accompanied by metabolic disorders. These findings may yield pharmacological strategies to block the cycle of obesity and its associated metabolic complications.

## Results

### Adipocyte-specific TRPM7 deficiency attenuates HFD-induced obesity

**Fig. 1:**
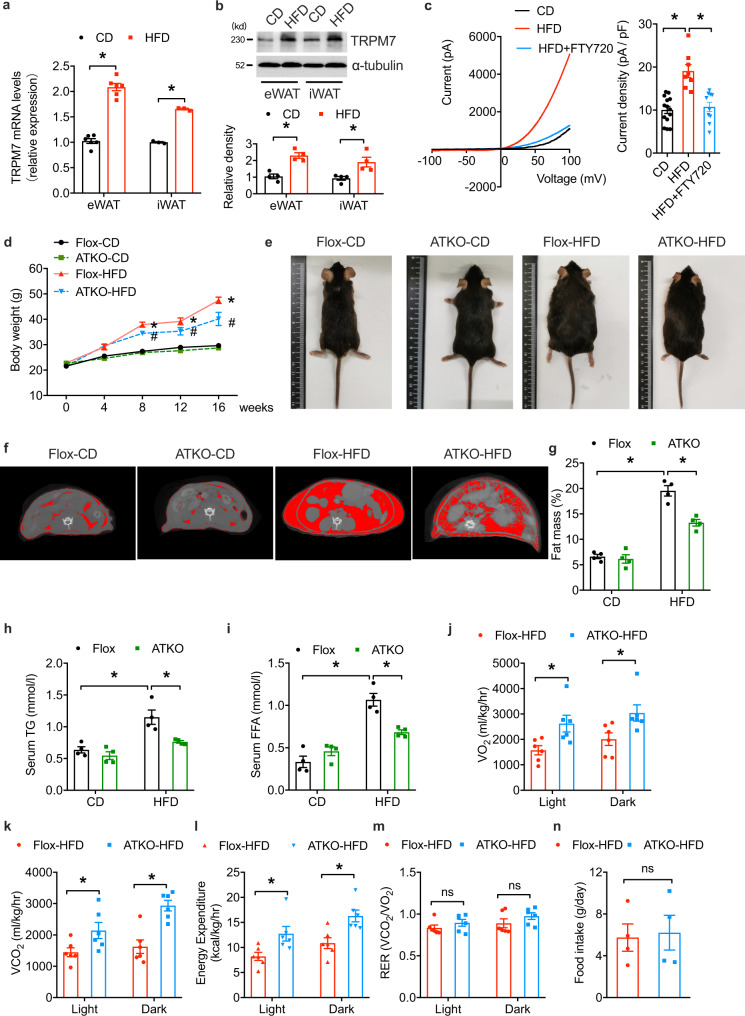
Adipocyte-specific TRPM7 ablation attenuates HFD-induced obesity. **a** TRPM7 mRNA level of eWAT and iWAT from mice after chow diet or HFD for 16 weeks (*n* = 6 mice, for eWAT, *n* = 3 mice for iWAT). **b** Western blot analysis of TRPM7 protein expression in adipose tissue of mice fed with chow diet and HFD for 16 weeks (*n* = 4 mice). **c** A representative I-V relationship of whole-cell TRPM7-mediated currents in freshly isolated adipocytes from mice after chow diet or HFD for 16 weeks. The statistics of TRPM7 current densities are shown in bar chart (*n* = 14 cells for CD, *n* = 8 cells for HFD, *n* = 10 cells for HFD + FTY720, from 4 mice). **d** Adipose-specific TRPM7 deletion (ATKO) mice and TRPM7^fl/fl^ (Flox) mice were fed with chow diet (CD) or high-fat diet (HFD) for 16 weeks and then body weight was measured (*n* = 5 mice), *comparison of Flox-HFD vs. Flox-CD, ^#^comparison of ATKO-HFD vs. Flox-HFD. **e** Representative photographs of Flox and ATKO mice fed with CD or HFD (*n* = 4 mice). **f** Representative micro-CT images of abdominal fat (*n* = 4 mice). **g** Fat mass analysis of Flox and ATKO mice fed with CD or HFD (*n* = 4 mice). **h**, **i** Serum triglycerides (TG) (**h**) and free fat acid (FFA) (**i**) in Flox and ATKO mice fed with CD or HFD (*n* = 4 mice). **j**–**m** Metabolic cage analysis of CD- and HFD-fed Flox and ATKO mice to measure oxygen consumption (VO_2_) (**j**), carbon dioxide production (VCO_2_) (**k**), energy expenditure (EE) (**l**), and respiratory exchange rate (RER) (**m**) (*n* = 6 mice). **n** Daily food take in Flox and ATKO mice fed with HFD (*n* = 4 mice). All statistical data were assessed using 2-tailed Student’s *t* test (**a**, **b**, **j**–**n**) or one-way ANOVA (**c**, **d**, **g**–**i**) and are presented as mean ± SEM. *p < 0.05. Source data are provided as a Source Data file. kd, relative molecular weight in kilodalton; ns, not significant.

To evaluate the function of TRPM7 in adipose tissue, we explored TRPM7 expression in adipose tissue in obesity. We measured the protein and mRNA levels of TRPM7 in visceral epididymal WAT (eWAT) and subcutaneous inguinal WAT (iWAT). mRNA and protein levels of TRPM7 were increased in the eWAT and iWAT induced by an HFD, compared to their controls (Fig. 1a, b). We measured TRPM7 channel activity in voltage-ramp mode in freshly dispersed eWAT adipocytes. We observed that an HFD potentiated the whole-cell current, which was almost completely inhibited by the addition of the TRPM7 antagonist FTY720, compared to the corresponding chow diet (CD)-fed controls (Fig. 1c). Thus, the expression and activity of TRPM7 appeared to be positively associated with obesity in mice. The dynamics of TRPM7 expression led us to investigate whether TRPM7 regulates metabolic dysfunction in adipose tissues and the development of obesity.

To specifically assess the role of TRPM7 in adipose tissue, we crossbred TRPM7^fl/fl^ (Flox) mice with Adipoq-Cre mice to generate adipose-specific TRPM7 knockout mice (ATKO) (Supplementary Fig. 1a, b). Supplementary Fig. 1 shows the genotyping and expression assessment. As expected, TRPM7 protein and mRNA levels were decreased in adipose tissue (Supplementary Fig. 1c, d), but not in the skeletal muscle, spleen, brain, or heart (Supplementary Fig. 1e). TRPM7 expression was remarkably decreased in ATKO versus Flox mouse mature adipocytes (Supplementary Fig. 1f), and the stromal vascular fraction (SVF) TRPM7 levels did not differ (Supplementary Fig. 1g). Consistently, the TRPM7-mediated current was decreased by ~85% in mature adipocytes isolated from the adipose tissue of the TRPM7 ATKO mice (Supplementary Fig. 1h), reflecting the complete reduction of TRPM7 expression in adipocytes and the residual protein present in other cells of adipose tissue.

Adipocyte-specific TRPM7 knockout mice did not exhibit overt abnormalities. In mice fed a CD, the TRPM7 knockout did not affect body weight, fat weight, or fat distribution (Fig. 1d–g, and Supplementary Fig. 2). However, after 16 weeks of the experimental diet, TRPM7-deficient mice gained less weight and tended to be leaner than the controls when fed an HFD (Fig. 1d, e). The individual adipose tissue depot was scanned by micro-CT to determine the effect of TRPM7 loss on the decrease in adipose tissue, which may have contributed to the body weight reduction. Consistent with the weight reduction shown in Fig. 1d, e, micro-CT images illustrated dramatic decreases in adipose mass by TRPM7 knockout in the adipose tissues (Fig. 1f, g). The eWAT and iWAT weights were decreased in TRPM7-deficient mice challenged with an HFD compared to the controls, with no significant change in the brown adipose tissue (BAT) (Supplementary Fig. 2). We detected downregulation of triglycerides (TG) and free fatty acids (FFA) in the ATKO mice (Fig. 1h, i) compared to Flox mice fed with HFD.

The effects of TRPM7 ablation in adipose tissue on basic metabolic activity were also evaluated. TRPM7 deletion increased oxygen consumption (Fig. 1j and Supplementary Fig. 3a), carbon dioxide production (Fig. 1k and Supplementary Fig. 3b), and energy expenditure rate (Fig. 1l and Supplementary Fig. 3c) but had no significant effect on the respiratory exchange ratio (VCO_2_/VO_2_) (Fig. 1m and Supplementary Fig. 3d). Notably, weight loss in the HFD-fed ATKO mice was not related to the changes in food intake compared to the HFD-fed Flox mice (Fig. 1n), suggesting that the resistance of adipocyte-specific TRPM7 knockout mice to diet-induced obesity was attributed to higher catabolic rates.

### Adipose-specific TRPM7 deletion protects against HFD-induced systemic insulin resistance

**Fig. 2:**
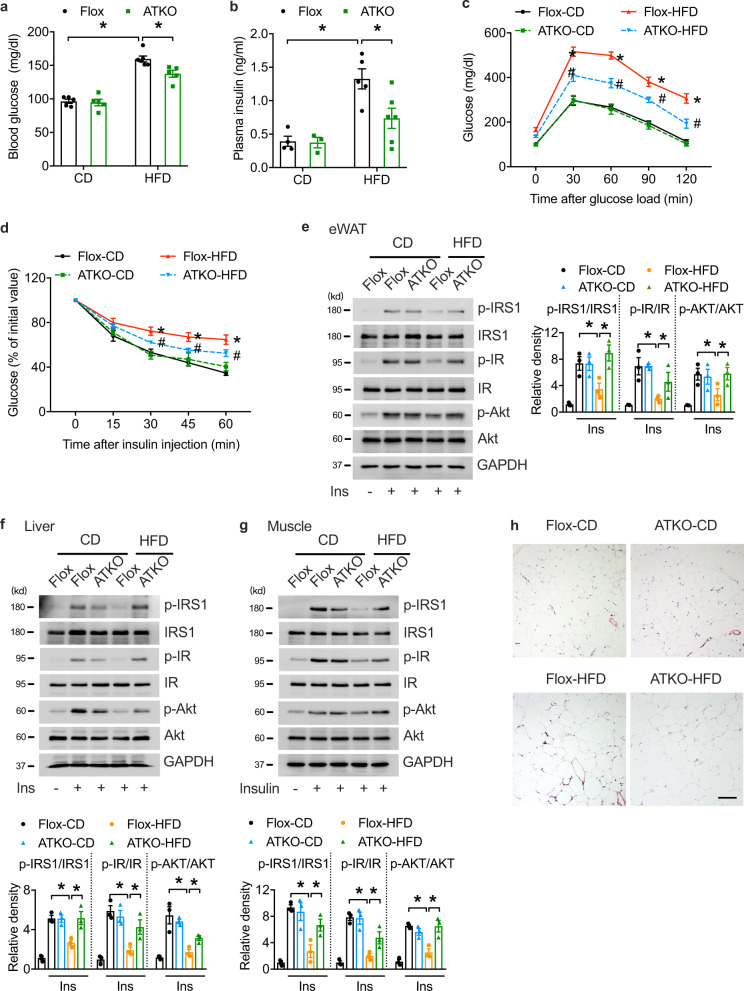
Adipocyte TRPM7 is required for glucose homeostasis and insulin sensitivity in vivo. **a**, **b** Serum glucose (**a**, n = 5 mice for Flox-CD, ATKO-CD, ATKO-HFD, *n* = 6 mice for Flox-HFD) and insulin (**b**, *n* = 4 mice for Flox-CD, *n* = 3 mice for ATKO-CD, *n* = 5 mice for Flox-HFD, *n* = 6 mice for ATKO-HFD) levels in overnight-fasted Flox and ATKO mice fed with CD or HFD. **c**, **d** Glucose tolerance test (**c**, *n* = 6 mice for Flox-CD, ATKO-CD, Flox-HFD, *n* = 5 mice for ATKO-HFD) and insulin tolerance test (**d**, *n* = 5 mice) in Flox and ATKO mice fed with CD or HFD, *comparison of Flox-HFD vs. Flox-CD, ^#^comparison of ATKO-HFD vs. Flox-HFD. **e**–**g** Immunoblot (IB) analysis for IRS-1 phosphorylation at Tyr^612^, IR phosphorylation at Tyr^1345^ and Akt phosphorylation at Thr^473^, in eWAT (**e**), liver (**f**), muscle (**g**) from Flox and ATKO mice fed with CD or HFD after i.p. injection of 0.75 U/kg insulin (*n* = 3 mice). **h** H&E staining of eWAT (scale bar, 100 μm) of Flox and ATKO mice fed with CD or HFD (*n* = 3 mice). All statistical data were assessed using one-way ANOVA and are presented as mean ± SEM. *p < 0.05. Source data are provided as a Source Data file. kd, relative molecular weight in kilodalton.

Given that obesity is closely associated with glucose intolerance and insulin resistance, we assessed glucose homeostasis in the CD- and HFD-fed control and TRPM7 ATKO mice. When fed with CD, there were no differences in fasting glucose, fasting insulin, and glucose or insulin tolerance among the genotypes (Fig. 2a–d). Compared to CD-fed mice, the HFD-fed Flox mice showed increased basal glucose and insulin levels, which were reduced in TRPM7 ATKO mice (Fig. 2a, b). Obese TRPM7 ATKO mice showed improved glucose and insulin tolerance compared to TRPM7 Flox littermates (Fig. 2c, d). To gain a better understanding of the role of TRPM7 in insulin resistance, we measured ex vivo insulin action. The phosphorylation of insulin-stimulated IRS-1, IR, and Akt was examined in the eWAT, liver, and skeletal muscle of mice. Insulin-stimulated IRS-1/IR/Akt cascade activation was significantly higher in the adipose tissue, liver, and skeletal muscle of the TRPM7 knockout mice, indicating that TRPM7 ablation in adipocytes prevents HFD-induced insulin resistance in adipose tissue and leads to increased insulin sensitivity in the liver and skeletal muscle under HFD (Fig. 2e–g).

To store excess energy as triglycerides in obesity, the adipose tissue expands through hypertrophy of the existing adipocytes. Thus, we studied the adipocyte architecture of obese TRPM7 ATKO and Flox mice. The adipocytes in the TRPM7 ATKO mice were smaller than those in the Flox mice (Fig. 2h), and showed a lower average cell size than those in the controls (Supplementary Fig. 4a). Adipocyte frequency analysis in the visceral adipose tissue illustrated that the TRPM7 ATKO mice had a reduced frequency of large adipocytes and a higher frequency of small adipose cells (Supplementary Fig. 4b). We also assessed the effect of adipose TRPM7 deletion on hepatic and BAT lipid deposition. Histological and oil red O staining analysis of the liver showed that the number and size of lipid droplets were smaller in the TRPM7 ATKO mice than in the Flox mice fed an HFD (Supplementary Fig. 4c). TRPM7 knockdown also decreased lipid storage in the BAT of the HFD-fed mice (Supplementary Fig. 4d). These findings revealed that TRPM7 impairs adipocyte function along with systemic metabolic homeostasis, and adipose-specific TRPM7 deletion improves insulin sensitivity due to the restored function of the adipocytes.

### Adipocyte-specific TRPM7 knockout mice have attenuated adipose inflammation induced by HFD

**Fig. 3:**
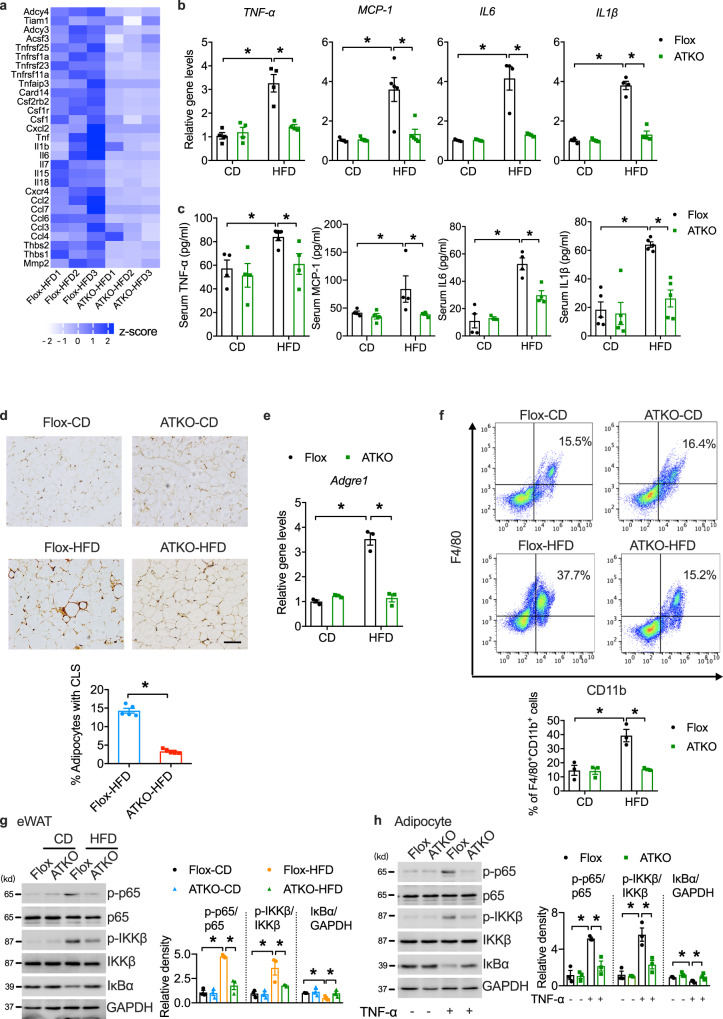
Adipocyte-specific TRPM7 knockout inhibits adipose inflammation in obese mice. **a** Gene expression profile was compared between eWAT derived from Flox and TRPM7 ATKO mice in response to HFD treatment. The heat map was generated based on expression of the significantly changed genes related to inflammatory responses (blue, upregulated; white, downregulated) (*n* = 3 mice). **b** Relative mRNA expression of pro-inflammatory cytokines in eWAT (*n* = 4 mice for *TNF-α*, *IL6* and *IL1β*, *n* = 5 mice for *MCP-1*). **c** Serum circulating levels of TNF-α (*n* = 4 mice for Flox-CD, ATKO-CD, ATKO-HFD, *n* = 5 mice for Flox-HFD), MCP-1 (*n* = 4 mice), IL6 (*n* = 4 mice for Flox-CD, Flox-HFD, ATKO-HFD, *n* = 3 mice for ATKO-CD) and IL1β (*n* = 5 mice). **d** F4/80 staining of eWAT (scale bar, 100 μm**)** from Flox and ATKO mice fed with CD or HFD. Bar chart showing density of crown-like structure (CLS) (*n* = 5 mice). **e** Relative mRNA levels of macrophage marker *Adgre1* (*F4/80*) in eWAT (*n* = 3 mice). **f** FACS analysis of F4/80^+^/CD11b^+^ cells in stromal vascular fraction (SVF) from eWAT of Flox and ATKO mice fed with CD or HFD (*n* = 3 mice). **g** Western blots of the indicated total and phosphor-proteins in whole tissue lysates prepared from eWAT of Flox and ATKO mice fed with CD or HFD (*n* = 3 mice). **h** Western blots analysis of the indicated proteins in cultured primary adipocytes from Flox and ATKO mice stimulation with TNF-α (60 ng/ml) for 15 min (*n* = 3 mice). All statistical data were assessed using one-way ANOVA (**b**, **c**, **e**–**h**) or 2-tailed Student’s *t* test (**d**) and are presented as mean ± SEM. *p < 0.05. Source data are provided as a Source Data file. kd, relative molecular weight in kilodalton.

Exaggerated adipose inflammation and macrophage recruitment contribute to glucose intolerance and insulin resistance. The global gene expression profiles of eWAT in HFD-fed Flox and TRPM7 ATKO mice were analyzed by RNA sequencing (RNA-seq). Based on our RNA-seq data, in the TRPM7 ATKO mice, genes involved in inflammation, such as *MCP-1*, *IL6*, *IL1β*, *IL18, TNF-α*, and *Cxcr4*, were downregulated (Fig. 3a). Consistent with RNA-seq results, quantitative polymerase chain reaction (qPCR) verified these changes in the eWAT of the TRPM7 ATKO mice (Fig. 3b). Furthermore, adipocyte depletion led to a significant reduction in circulating pro-inflammatory factors (TNF-α, MCP-1, IL6, and IL1β) (Fig. 3c).

Staining of macrophage marker F4/80 (Adgre1) in the eWAT showed fewer crown-like structures (CLS), which are formed by adipose macrophage recruitment, in the TRPM7 ATKO mice (Fig. 3d). Consistently, HFD-fed TRPM7 ATKO mice demonstrated a marked reduction in *Adgre1* expression (Fig. 3e) and in the number of F4/80^+^CD11b^+^ macrophages in the stromal vascular fractions (SVFs) of eWAT, as determined by fluorescence-activated cell sorting (FACS) analysis (Fig. 3f). Lean TRPM7 ATKO mice showed the similar pattern in macrophage infiltration compared to Flox littermates (Fig. 3d–f).

As canonical IKKβ/NF-κB signaling, which is activated by HFD challenge, is required for inflammatory gene induction, we next examined the integrity of this pathway in the HFD-fed TRPM7 ATKO mice. HFD treatment induced the phosphorylation of IKKβ, NF-κB p65, and downregulated IκBα level in the eWAT of the Flox mice; however, these effects were significantly alleviated by TRPM7 knockout (Fig. 3g). To determine whether the hypoactivation of NF-κB signaling caused by TRPM7 knockout was cell-autonomous, we prepared epididymal adipocyte cultures from the Flox and ATKO mice. Upon complete differentiation, adipocytes were treated with TNF-α and processed for the analysis of the NF-κB signaling pathway and related pro-inflammatory gene expression. TRPM7 knockdown in TRPM7 ATKO adipocytes significantly reduced TNF-α-stimulated phosphorylation of IKKβ, NF-κB p65, and IκBα degradation (Fig. 3h), and lowered the gene expression of *MCP-1*, *IL6*, and *IL1β*, and MCP-1 secretion when compared to their respective control adipocytes (Supplementary Fig. 5a, b). In vitro, macrophage chemotaxis toward conditional medium from TRPM7 knockout adipocytes was markedly reduced compared to Flox stimulated by TNF-α (Supplementary Fig. 5c).

To ensure our results were not affected by body weight loss in TRPM7 knockout mice, we performed the acute deletion of adipocyte TRPM7 that did not influence whole-body weight. To address this question, we depleted TRPM7 in eWAT using an approach of adeno-associated virus (AAV) (Supplementary Fig. 6a). Flox mice fed HFD for 12 weeks were given AAV injection (AAV-Vec, TRPM7^eWAT WT^, or AAV-Adipoq-Cre, TRPM7^eWAT KO^) in both sides of eWAT. At 4 weeks after AAV injection, mice were sacrificed for further tissue analysis. As expected, TRPM7 mRNA expression level was reduced significantly in isolated adipocytes (Supplementary Fig. 6b). The decrease of TRPM7 was limited to adipocytes and were not observed in SVFs (Supplementary Fig. 6b). No change of body weight and fasting blood glucose level was found between two groups (Supplementary Fig. 6c–e). Parameters such as plasma insulin levels and eWAT weights in TRPM7^eWAT KO^ mice were decreased as compared with WT mice, but there was no statistical difference between two groups (Supplementary Fig. 6f–h). In parallel, glucose tolerance was improved by TRPM7 depletion with no significant difference (Supplementary Fig. 6i). Moreover, this acute deletion of adipocyte TRPM7 led to reduced macrophage infiltration (Supplementary Fig. 6j) and lower expression of inflammation marker genes (Supplementary Fig. 6k) in eWAT compared with controls. NF-κB signaling activity was reduced (Supplementary Fig. 6l) and insulin response was largely improved (Supplementary Fig. 6m) in TRPM7-deficient eWAT. Adipocyte-specific TRPM7 knockout using AAV-Cre transduction in eWAT obtained similar results in the Adipoq-Cre TRPM7 knockout model, strongly supporting our conclusions.

Collectively, our data showed that adipocyte-specific TRPM7 deletion decreased pro-inflammatory cytokine secretion and inhibited pro-inflammatory macrophage infiltration in adipose tissue, causing attenuated inflammatory responses and contributing to improved insulin action.

### TRPM7-dependent-Ca^2+^ influx regulates the NF-κB cascade to induce adipose inflammation

**Fig. 4:**
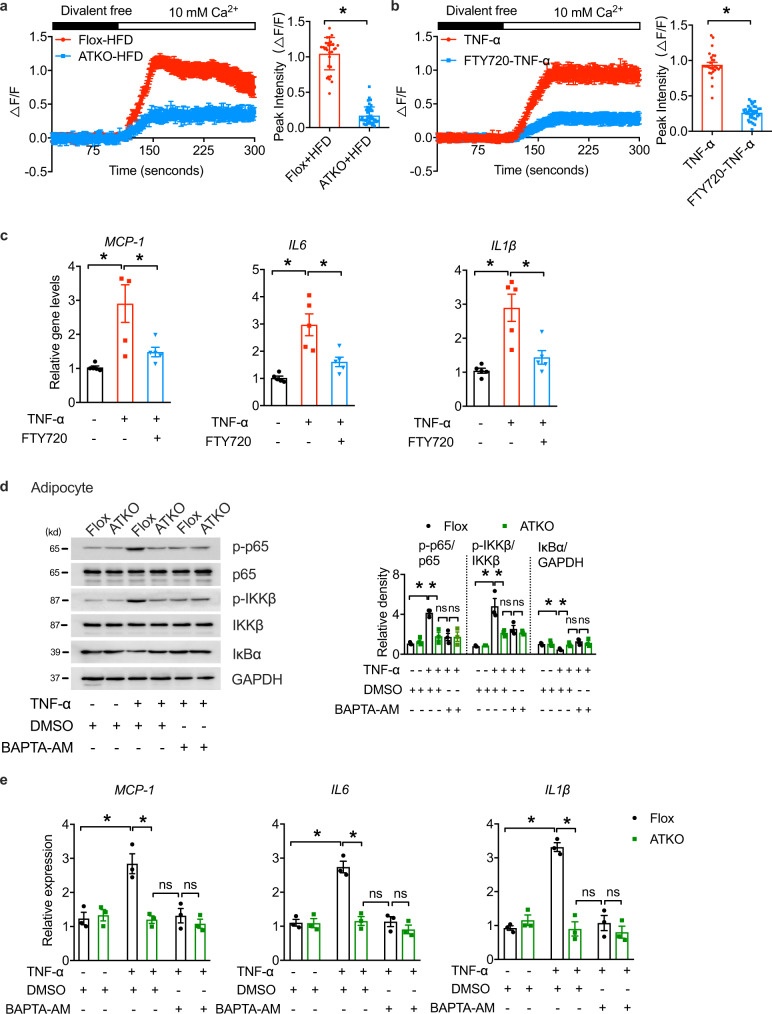
Ca2+ elevations are responsible for TRPM7-dependent adipocyte inflammation. **a** Relative changes in the fluorescence of Fluo-4-loaded mature adipocytes isolated from eWAT of Flox and ATKO mice fed with HFD, reflecting changes in intracellular Ca^2+^ concentration ([Ca^2+^]_i_). Bar chart showed the quantification of peak [Ca^2+^]_i_. These represent typical results from three independent experiments (*n* = 30 cells for Flox-HFD, *n* = 45 cells for ATKO-HFD, from 3 mice). **b** Relative changes in [Ca^2+^]_i_ over time in mature adipocyte pretreated with FTY720 (5 μM) for 15 min, followed by stimulation with TNF-α (60 ng/ml) for 15 min as indicated. Bar chart showed the quantification of peak [Ca^2+^]_i_. Results are a compilation of three experiments (*n* = 27 cells for TNF-α, *n* = 38 cells for FTY720-TNF-α, from 3 different experiments). **c** qPCR analysis of indicated gene expression of *MCP-1*, *IL6*, and *IL1β* in differentiated adipocytes treated as depicted. Adipocytes were treated with TNF-α (40 ng/ml; 12 h) with FTY720 (5 μM; 15 min) pretreatment (*n* = 5 biologically independent experiments). **d** Western blots of phosphorylated and total p65, IKKβ, IκBα and GAPDH in cultured primary adipocytes from eWAT of Flox and ATKO mice. Prior to TNF-α treatment (60 ng/ml; 15 min), adipocytes were pretreated with DMSO or BAPTA-AM (10 μM) for 30 min in serum-free media (*n* = 3 biologically independent experiments). **e** Relative mRNA expression of indicated inflammatory genes in cultured primary adipocytes from Flox and ATKO mice. Cells were treated with BAPTA-AM (10 μM; 30 min) or DMSO as indicated prior to TNF-α treatment (60 ng/ml; 15 min) (*n* = 3 biologically independent experiments). Statistical data were assessed using two-tailed Student’s *t* test (**a**, **b**) or one-way ANOVA statistical analysis (**c**–**e**) and are presented as mean ± SEM. *p < 0.05. Source data are provided as a Source Data file. kd, relative molecular weight in kilodalton.

The potent regulatory activity of TRPM7 in adipose inflammation and its associated metabolic dysfunction prompted us to explore the underlying mechanisms. Considering the role of [Ca^2+^]_i_ rise in adipocyte function related to insulin sensitivity^11^, and the capacity of TRPM7-mediated Ca^2+^ entry to modulate inflammation in other cell types^25,29^, we hypothesized that TRPM7 regulates adipocyte inflammation by controlling Ca^2+^ influx under an HFD. To do this, freshly isolated mature adipocytes were perfused with divalent-free solution (DVF, Ca^2+^, and Mg^2+^-free) for 4 min, after which the extracellular bath solution was replaced with a Ca^2+^-containing solution. Compared to Flox adipocytes from HFD-fed mice, TRPM7 knockout adipocytes exhibited significantly diminished [Ca^2+^]_i_ elevations; the mean peak [Ca^2+^]_i_ level was down to ~25% of that of the corresponding controls (Fig. 4a). In addition, we observed a robust rise in cytosolic [Ca^2+^]_i_ in adipocytes after stimulation with TNF-α, while FTY720, a TRPM7 channel blocker, decreased TNF-α-evoked Ca^2+^ influx compared to that in untreated cells (Fig. 4b). Therefore, we concluded that TRPM7 controls Ca^2+^ entry triggered by TNF-α and HFD.

The use of FTY720 mimicked the effects of TRPM7 deletion on inhibiting the transcription of *MCP-1*, *IL6* and *IL1β* in cells responding to TNF-α (Fig. 4c), suggesting that TRPM7 channel activity plays a critical role in TNF-α-induced gene transcription. BAPTA-AM, a high-affinity Ca^2+^ chelator used to clamp and deplete intracellular Ca^2+^, prevented TNF-α-induced [Ca^2+^]_i_ elevation, which, in turn, reduced the activity of the NF-κB-IKKβ pathway in the control adipocytes but had little additional effects on the TRPM7 knockout cells (Fig. 4d). BAPTA-AM treatment also mimicked the effects of FTY720 on the NF-κB-dependent gene transcripts (Fig. 4e).

To dissect the role of TRPM7 kinase, we investigated the influence of a TRPM7 kinase dead mutant on NF-κB pathway. TRPM7 knockout adipocytes were transfected with TRPM7 adenovirus (TRPM7 wild type, Ad-TRPM7^WT^) or TRPM7 kinase dead mutant adenovirus (K1646A, Ad-TRPM7^KA^), and their control adenovirus (Ad-Vec) respectively (Supplementary Fig. 7). While both Ad-TRPM7^WT^ and Ad-TRPM7^KA^ exhibited a significant restoration in inflammatory response to TNF-α, there was no significant difference between the two groups (Supplementary Fig. 7a, b), suggesting that the lack of TRPM7 channel function rather than its kinase activity accounts for adipose inflammation. As TRPM7 transports Mg^2+^ other than Ca^2+^, we tested the function of Mg^2+^ in adipocytes. As expected, Mg^2+^ influx was also inhibited by FTY720 (Supplementary Fig. 8a). Of note, pretreatment of adipocytes with Ca^2+^ chelators (BAPTA-AM and EGTA) or chelator of both Mg^2+^ and Ca^2+^ (EDTA) did not exert response to TNF-α stimulation in ATKO adipocytes transfected with adenovirus expressing TRPM7 (Supplementary Fig. 8b, c). Interestingly, TNF-α-induced inflammatory genes expression and NF-κB cascade activation was monitored only in the presence of excess Ca^2+^ over EDTA, but not Mg^2+^ (Supplementary Fig. 8b,c), thus further supporting the notion that elevated [Ca^2+^]_i_ alters the inflammatory response in adipocytes. Overall, these results indicated that TRPM7 channel conducts Ca^2+^ signal to mediate NF-κB signaling.

### TRPM7-mediated adipocyte inflammation depends on TAK1 activation

**Fig. 5:**
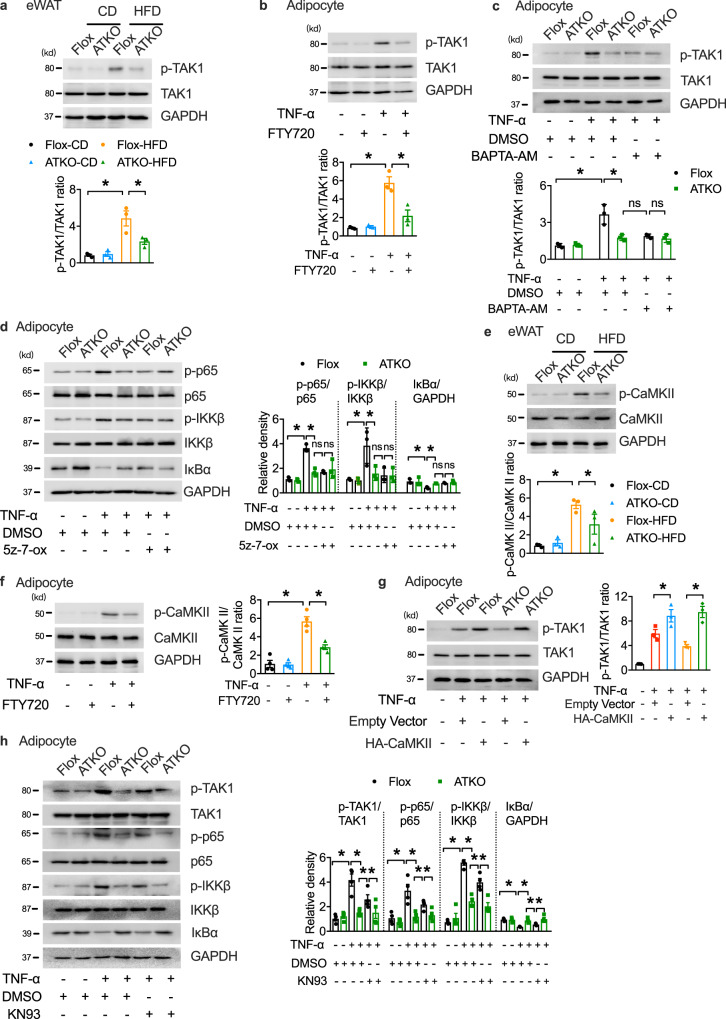
TRPM7 promotes NF-κB signal by TAK1 activation partly dependent on CaMKII. **a** Western blots of phosphorylation of TAK1 in eWAT from CD- and HFD- fed Flox and ATKO mice (*n* = 3 mice). **b** Western blot analysis of TAK1 phosphorylation in differentiated adipocytes pretreated with FTY720 (5 μM) for 15 min, and then treated with TNF-α (60 ng/ml) for 15 min as indicated (*n* = 3 biologically independent experiments). **c** Immunoblot analysis of TAK1 phosphorylation in cultured primary adipocytes treated with BAPTA-AM (10 μM; 30 min) followed by TNF-α administration (60 ng/ml; 15 min) (*n* = 3 biologically independent experiments). **d** Immunoblot analysis of adipocytes treated with TNF-α, DMSO or 5z-7-ox. Prior to TNF-α treatment (60 ng/ml; 15 min), adipocytes were pretreated with 5z-7-ox (100 nM) for 30 min (*n* = 3 biologically independent experiments). **e** Immunoblot analysis of p-CaMKII and total CaMKII in eWAT of indicated mice (*n* = 3 mice). **f** Phosphorylated and total CaMKII in FTY720 (5 μM; 15 min) loaded adipocytes in response to TNF-α (60 ng/ml; 15 min) were assessed by western blotting (*n* = 4 biologically independent experiments). **g** Western blot of phosphorylation of TAK1 in 3T3-L1 adipocytes transfected with HA-CaMKII or empty vector (*n* = 3 biologically independent experiments). **h** The levels of proteins in mouse cultured primary adipocytes treated with KN93 (10 μM; 30 min) or DMSO followed by TNF-α administration (60 ng/ml; 15 min) (*n* = 4 biologically independent experiments). All Statistical data in Fig. 5 were assessed using one-way ANOVA and are presented as mean ± SEM. *p < 0.05. Source data are provided as a Source Data file. kd, relative molecular weight in kilodalton.

TAK1, a crucial upstream factor in NF-κB signaling, phosphorylates IKKβ and elicit IκBα phosphorylation and degradation to induce NF-κB activation^3^. We measured the role of TAK1 activation in modulating the TRPM7-dependent NF-κB cascade. HFD feeding significantly increased TAK1 Thr 187 phosphorylation in the adipose tissue, and TRPM7 deficiency abrogated the promotion of TAK1 activation in eWAT by HFD (Fig. 5a). We also found that TRPM7 inhibition downregulated TNF-α-induced TAK1 phosphorylation in differentiated cultured primary adipocytes (Fig. 5b). BAPTA-AM inhibited TNF-α-induced TAK1 phosphorylation in TRPM7 wild-type adipocytes but had little additional effect on the TRPM7 knockout adipocytes (Fig. 5c). Next, we measured the NF-κB pathway activity in adipocytes pretreated with the TAK1 inhibitor (5z)−7-oxozeaenol (5z-7-ox). As shown in Fig. 5d, activation of the NF-κB pathway was greatly compromised in the TRPM7 knockout adipocytes, and pharmacological inhibition of TAK1 using 5z-7-ox also attenuated activation of the NF-κB cascade in the adipocytes from the Flox mice, but had no more notable effects on the TRPM7 knockout adipocytes. Gene expression analysis illustrated that 5z-7-ox did not further inhibit gene expression of *MCP-1*, *IL6*, and *IL1β* in the TRPM7 knockout cells (Supplementary Fig. 9a), which was consistent with the observed reduction in NF-κB signaling.

Next, we explored the potential link between TRPM7-mediated Ca^2+^ signal and TAK1 activation. Calcium/calmodulin-dependent protein kinase II (CaMKII), a ubiquitous serine/threonine protein kinase, is activated by Ca^2+^ influx^30^. Upon an increase in calcium levels, Ca^2+^/calmodulin binding to CaMKII relives autoinhibition and evokes CaMKII autophosphorylation, which leads to the persistent activation of kinase^30^. Previous studies have shown that TRPM7 mediates neuronal cell death through Ca^2+^-CaMKII signaling^31^. Moreover, CaMKII activation promotes obesity-induced metabolic syndrome^32^, and CaMKII, as an upstream factor of TAK1, directly binds and phosphorylates TAK1^33–35^. We detected p-CaMKII downregulation in the eWAT of HFD-fed TRPM7 knockout mice (Fig. 5e). FTY720 blocked CaMKII activity in the cultured primary adipocytes in response to TNF-α (Fig. 5f). Moreover, TNF-α-stimulated phosphorylation of TAK1 in adipocytes was strongly enhanced by CaMKII (Supplementary Fig. 9b). CaMKII overexpression reversed the inhibitory effect of the TRPM7 knockout-induced depression of TAK1 activity upon TNF-α treatment (Fig. 5g).

Although the CaMKII inhibitor KN93 suppressed TNF-α-induced TAK1 phosphorylation, the level of inhibition was incomplete compared to TRPM7 knockdown (Fig. 5h). The ability of KN93 to partially block TAK1 activation was also supported by the measurement of NF-κB signaling activity and downstream gene expression (Fig. 5h and Supplementary Fig. 9c). These results suggest that TAK1 is indispensable for TRPM7-mediated adipose inflammation, and that CaMKII directly, but partially, contributes to TRPM7-dependent TAK1 phosphorylation, indicating that other mechanisms in addition to CaMKII activation are involved.

### TRPM7-dependent TAK1 phosphorylation requires both CaMKII and TRAF6

**Fig. 6:**
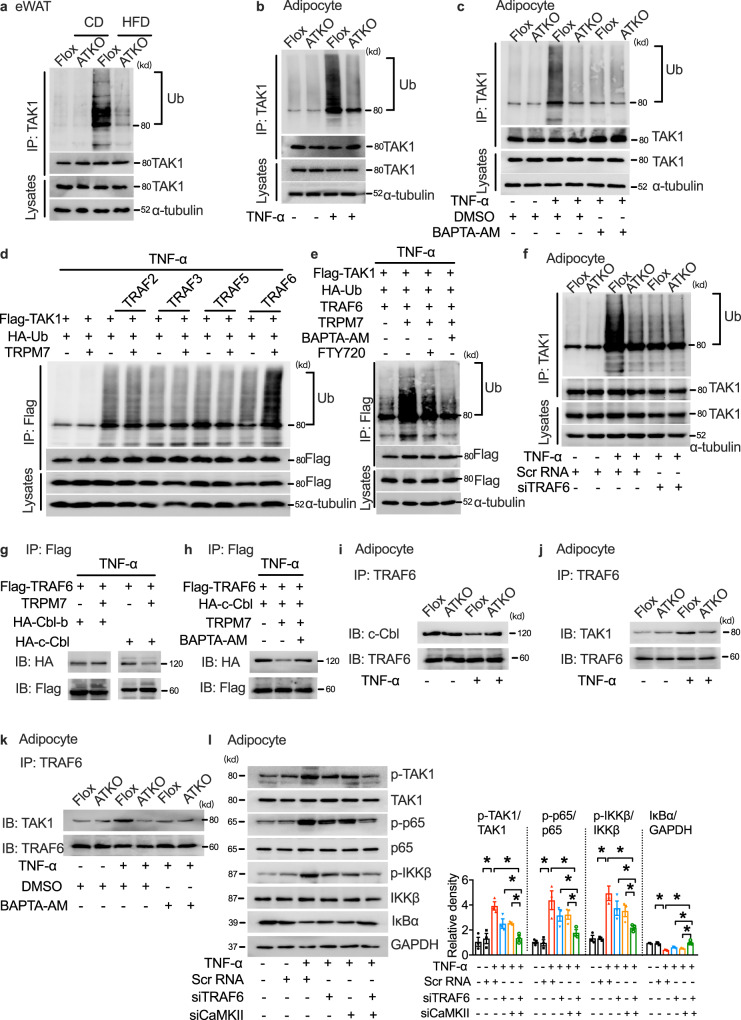
E3 ligase TRAF6 functions as co-regulator of CaMKII in TRPM7-induced TAK1 activation. **a** Ubiquitination level of TAK1 in eWAT of Flox and ATKO mice in the presence or absence of HFD for 16 weeks (*n* = 3 mice). **b** The ubiquitination of TAK1 in TNF-α (60 ng/ml; 30 min)-stimulated adipocytes. **c** Immunoprecipitation (IP) analysis of TAK1 ubiquitination in cultured primary adipocytes pretreated with BAPTA-AM (10 μM; 30 min) and followed by TNF-α (60 ng/ml; 30 min). **d** IP and western blot of indicated proteins in HEK293T cells transfected with Flag-TAK1, HA-Ub and the indicated E3 ubiquitin ligases in the presence or absence of TRPM7 in the treatment of TNF-α (60 ng/ml; 30 min). **e** IP and western blot of indicated proteins in HEK293T cells transfected with Flag-TAK1, HA-Ub, TRAF6 and TRPM7 in response to either BAPTA-AM (10 μM; 30 min) or FTY720 (5 μM; 15 min) stimulation followed by TNF-α treatment (60 ng/ml; 30 min). **f** The level of TAK1 ubiquitination in cultured primary adipocytes transfected with TRAF6 siRNA upon TNF-α challenge (60 ng/ml; 30 min). **g** IB or IP lysates from HEK293T cells transfected with various plasmids as indicated. **h** Interaction of TRAF6 with c-Cbl in HEK293T cells transfected with Flag-tagged TRAF6 and HA-tagged c-Cbl in the presence or absence of TRPM7 or BAPTA-AM (10 μM; 30 min) prior to TNF-α treatment (60 ng/ml; 30 min). **i** Endogenous interaction of TRAF6 with c-Cbl in TNF-α (60 ng/ml; 30 min)-treated adipocytes. **j** Endogenous interaction of TRAF6 with TAK1 in TNF-α (60 ng/ml; 30 min)-treated adipocytes. **k** Co-immunoprecipitation of TRAF6 with TAK1 in cultured primary adipocytes in response to TNF-α (60 ng/ml; 30 min). **l** The level of key proteins of the NF-κB signaling cascade in TRAF6 siRNA, CaMKII siRNA or scramble siRNA transfected differentiated 3T3-L1 adipocytes followed by TNF-α treatment (60 ng/ml; 15 min). *n* = 3 biologically independent experiments for (**a**–**l**). Statistical data in (**l**) was assessed using one-way ANOVA and are presented as mean ± SEM. *p < 0.05. Source data are provided as a Source Data file. kd, relative molecular weight in kilodalton.

In addition to CaMKII regulation, ubiquitination-induced TAK1 catalytic activity results in autophosphorylation and enzymatic activation. Several studies have shown that an HFD challenge leads to TAK1 hyperactivation via TAK1 polyubiquitination and subsequent phosphorylation^36,37^. Considering the necessity of TAK1 polyubiquitination for TAK1 phosphorylation and IKK/NF-κB activation, we investigated whether TAK1 ubiquitination was involved in TRPM7-regulated adipocyte inflammation during metabolic disorders. TAK1 ubiquitination increased in the adipose tissue of obese mice and was ameliorated by TRPM7 depletion (Fig. 6a). The reduction in TAK1 ubiquitination by TRPM7 depletion was confirmed in TNF-α-challenged cultured primary adipocytes (Fig. 6b). Clamping [Ca^2+^]_i_ using BAPTA-AM completely ablated the ubiquitination of TAK1 induced by TNF-α but had little additional effect on TRPM7 knockout adipocytes (Fig. 6c).

Next, we aimed to identify the E3 ligase that mediates TAK1 ubiquitylation. We screened several E3 ligases that have been reported to influence TAK1 ubiquitination or affect the IKK/NF-κB cascade. Some members of the tumor necrosis factor receptor-associated factor (TRAF) family, including TRAF2, TRAF3, TRAF5, and TRAF6, contain the RING domain and act as E3 ubiquitin ligases to promote NF-κB activation^38^. TRAF3 has been reported to directly ubiquitinate TAK1^39^, and TRAF2 and TRAF6 promote the formation of TRAF2-TAK1-TAB or TRAF6-TAK1-TAB complexes to mediate K63-linked polyubiquitination of TAK1, leading to the activation of TAK1^40^. Thus, the four TRAF members were examined for their potential functional relevance in TAK1 ubiquitination. A ubiquitination assay revealed that TRAF2, TRAF3, TRAF5, and TRAF6 promoted TAK1 ubiquitination in response to TNF-α in HEK293T cells. Only TRAF6-mediated ubiquitination of TAK1 was dramatically strengthened upon TRPM7 overexpression (Fig. 6d). Both BAPTA-AM and FTY720 attenuated TRAF6-dependent TAK1 ubiquitination in TRPM7-overexpressed HEK293T cells (Fig. 6e). TRAF6 silencing markedly reduced TAK1 ubiquitination, whereas TRPM7 knockdown failed to show further inhibitory effects on TAK1 ubiquitination in cultured primary adipocytes (Fig. 6f). The RING domain ubiquitin ligase TRAF6 catalyzes the synthesis of lysine (Lys)−63 (K63)-linked polyubiquitin chains^41^. The formation of K63-linked polyubiquitin chains attached to TAK1 is followed by autophosphorylation at T187 and S192 and activation of endogenous TAK1^42,43^. By transfecting HEK293T cells with HA-tagged K63 only-ubiquitin (K63-Ub) or a K63R ubiquitin mutant (K63R-Ub), we observed that TRPM7 promoted K63-linked ubiquitination of TAK1 in response to TNF-α stimuli (Supplementary Fig. 10a).

To identify the potential molecular link between TRPM7-dependent-Ca^2+^ signal and the TRAF6-TAK1 cascade, we acquired protein-protein interaction data from BioGRID and the Human Protein Reference Database (HPRD). After filtering from BioGRID and HPRD, we identified two TRAF6-binding proteins, c-Cbl and Cbl-b, possessing the EF-hand domain and containing a classical motif implicated in Ca^2+^ binding, that might be candidates for TRAF6-induced TAK1 ubiquitination. A co-immunoprecipitation assay revealed that TRAF6-c-Cbl binding, but not TRAF6-Cbl-b binding, was inhibited by TRPM7 (Fig. 6g). BAPTA-AM abrogated the inhibitory effect of TRAF6-c-Cbl binding in TRPM7-overexpressed cells (Fig. 6h). We also examined the endogenous interaction between TAK1 and c-Cbl, along with TAK1 and TRAF6, in adipocytes following TNF-α treatment. TNF-α stimulation decreased the interaction between TRAF6 and c-Cbl, which was restored in TRPM7 knockdown adipocytes (Fig. 6i). Moreover, the increase in TRAF6-TAK1 binding was blocked by TRPM7 depletion (Fig. 6j). Clamping of intracellular Ca^2+^ attenuated TRPM7-dependent TRAF6-TAK1 binding (Fig. 6k). Thus, these data suggest that the binding of the EF-hand protein c-Cbl to TRAF6 is antagonized by TRPM7-induced Ca^2+^ signal, and that TAK1 displaces TRAF6 from c-Cbl in response to Ca^2+^ influx.

We also measured the effect of TRAF6 on TRPM7-regulated adipocyte inflammation. Western blotting indicated that silencing of TRAF6 only had partial effects on the phosphorylation of TAK1 along with NF-κB cascade activation (Supplementary Fig. 10b), whereas silencing of both CaMKII and TRAF6 strongly suppressed TNF-α-induced TAK1-NF-κB signaling pathway activation (Fig. 6l). Furthermore, the detection of the downstream gene expression of *MCP-1*, *IL-6*, and *IL-1β* further confirmed the co-regulatory function of TRAF6 and CaMKII in TAK1 cascade activation (Supplementary Fig. 10c). Consistent with these findings, silencing of CaMKII or TRAF6 individually resulted in only a partial improvement in adipocyte insulin sensitivity, whereas silencing of both genes together strongly restored insulin signal transduction (Supplementary Fig. 10d). These observations suggest that CaMKII and TRAF6 act differently but jointly to promote TRPM7-mediated TAK1 activation and adipocyte inflammation.

### TAK1 overexpression in the setting of overnutrition rescues adipose inflammation

**Fig. 7:**
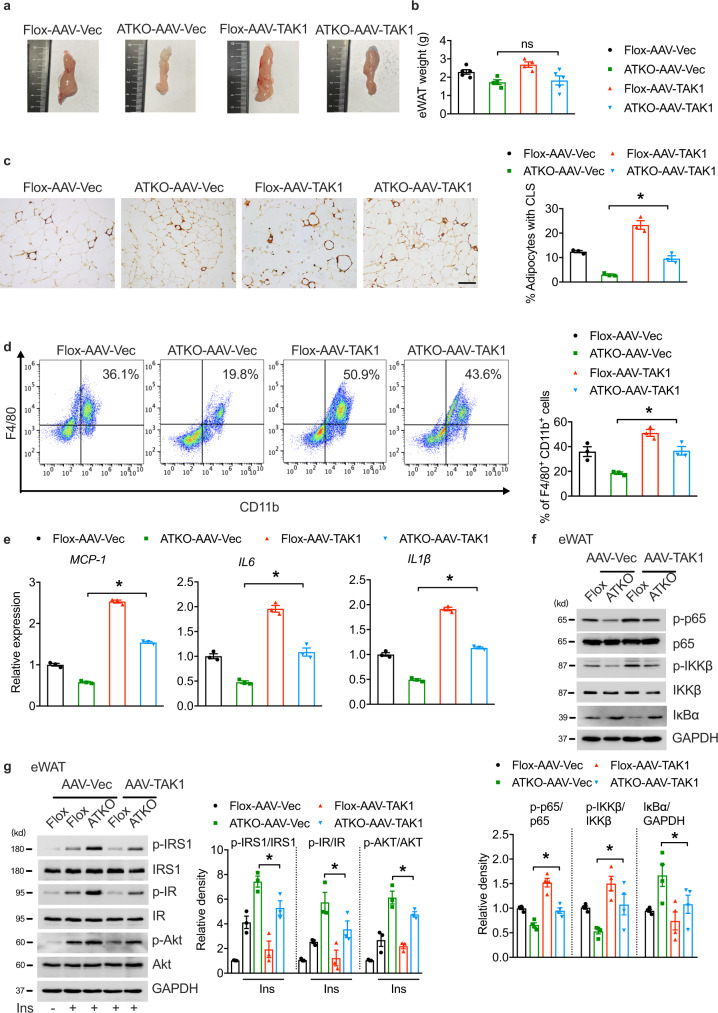
Adenovirus-mediated adipose TAK1 overexpression augments adipose inflammation in TRPM7 knockout mice fed with HFD. **a** Representative photographs of eWAT from HFD-fed Flox and ATKO mice injected with AAV-Vec or AAV-TAK1 (*n* = 3 mice). **b** The weight of eWAT injected with AAV-Vec or AAV-TAK1 from Flox and ATKO mice fed with HFD (*n* = 5 mice for Flox-AAV-Vec and ATKO-AAV-TAK1, *n* = 4 mice for ATKO-AAV-Vec and Flox-AAV-TAK1). **c** F4/80 staining of adipose tissue sections from the indicated groups (scale bar, 100 μm). Bar chart showed the density of CLS macrophages in eWAT (*n* = 3 mice). **d** FACS analysis of F4/80^+^ CD11b^+^ cells in SVF from eWAT of indicated mice. Bar chart showed the statistical analysis (*n* = 3 mice). **e** Pro-inflammatory genes expression in AAV-injected eWAT from the indicated mice (*n* = 3 mice). **f** The expression of proteins in NF-κB signaling cascade in the eWAT samples from the indicated groups (*n* = 4 mice). **g** Mice were fasted for 6 h, treated by injection with insulin (0.75 U/kg; i.p.), and total and phosphorylated IRS1, IR and Akt levels in the eWAT samples from the indicated groups were examined by immunoblot analysis (*n* = 3 mice). All statistical data were assessed using one-way ANOVA and are presented as mean ± SEM. *p < 0.05. Source data are provided as a Source Data file. kd, relative molecular weight in kilodalton; ns, not significant.

To examine the effects of TAK1 on TRPM7-mediated adipose inflammation in vivo, we used an adeno-associated virus (AAV) for sustained viral expression of full-length TAK1 in eWAT. After direct eWAT injection with either AAV-TAK1-Flag or AAV-Vec, both groups of mice were placed on an HFD prior to the analysis. A 3-fold increase in TAK1 gene expression was achieved after AAV injection into the fat pads, as confirmed by TAK1 protein expression using western blotting (Supplementary Fig. 11a, b). After 16 weeks of HFD, the weight did not differ between the TAK1 AAV-injected and Vec-AAV virus-injected fat pads in the TRPM7 knockout mice (Fig. 7a, b). Overexpression of TAK1 in eWAT had no effect on weight gain, fasting blood glucose, or fasting serum insulin levels under HFD conditions (Supplementary Fig. 11c–e). Notably, the glucose tolerance of TRPM7 knockout mice fed with an HFD was impaired slightly after TAK1 overexpression in eWAT, but with no statistical differences (Supplementary Fig. 11f), indicating that locally increasing TAK1 expression may not be sufficient to rescue the complete impairment of whole-body glucose homeostasis in HFD-fed TRPM7 ATKO mice.

Histological analysis of F4/80 staining demonstrated that the number of CLSs was almost threefold in TAK1-overexpressed eWAT compared to the injection of a control virus into the epididymal fat pad in TRPM7-deficient mice (Fig. 7c). Consistently, FACS analysis showed that the TRPM7 knockout-induced decrease in the number of double-positive CD11b/F4/80 macrophages in the SVFs was significantly reversed by adipose tissue TAK1 overexpression (Fig. 7d). We then repeated the gene expression analysis and observed that the adipose tissue pro-inflammatory markers (*MCP-1*, *IL6*, and *IL1β*) in the HFD-fed TRPM7 knockout mice were markedly increased by TAK1 overexpression (Fig. 7e), which was accompanied by a recovery of NF-κB cascade activation (Fig. 7f). Consistent with this finding, TAK1 overexpression in adipose tissue mitigated IRS1-AKT axis activation, which was improved by TRPM7 knockdown after 16 weeks of HFD consumption (Fig. 7g). Undoubtedly, the NF-κB cascade activation and pro-inflammatory marker genes expression were restored, while insulin signal transduction was impaired in primary adipocytes isolated from TAK1-overexpressed adipose tissue in TRPM7 depleted mice (Supplementary Fig. 11g–j).

### Pharmacological inhibition of TRPM7 suppresses adipose inflammation and improves glucose tolerance

**Fig. 8:**
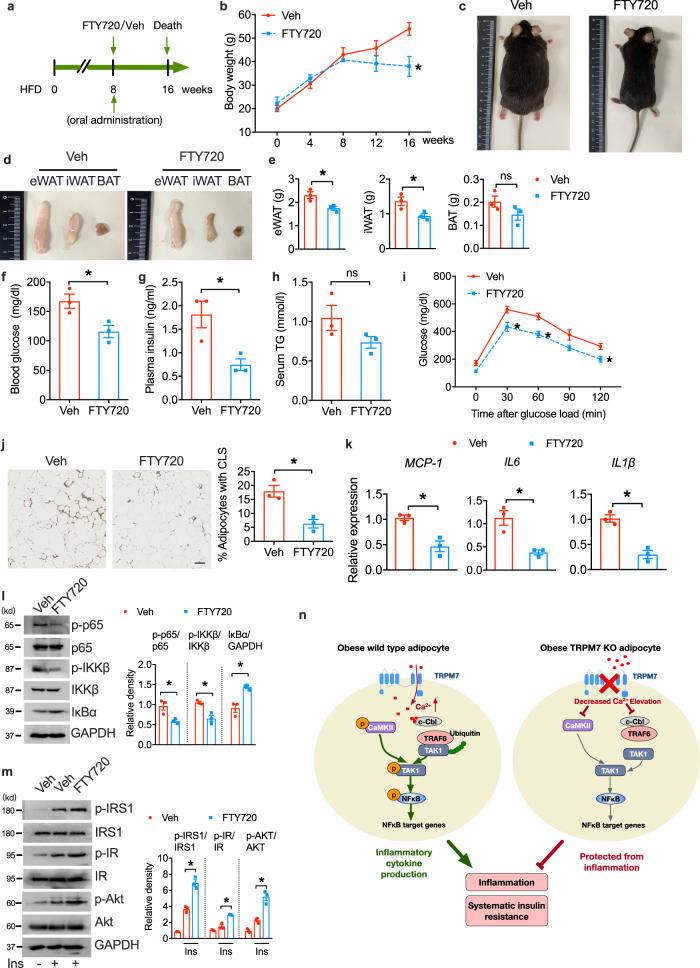
Effects of oral FTY720 on obese mice. **a** Schematic representation of experimental scheme for FTY720 oral administration. **b** Body weight on HFD (*n* = 3 mice). **c** Representative photographs of vehicle and FTY720-treated mice fed with CD or HFD (*n* = 3 mice). **d**, **e** Representative images (**d**) and fat mass weight (**e**) of eWAT, iWAT and BAT (*n* = 3 mice). **f**–**h** Serum glucose (**f**), insulin (**g**) and triglycerides (**h**) levels in overnight-fasted mice fed with HFD (*n* = 3 mice). **i** Glucose tolerance test in FTY720-treated and veh-treated mice fed with HFD (*n* = 3 mice). **j** Representative paraffin sections of eWAT stained for F4/80. Bar indicates 100 μm. Bar chart showing density of CLS (*n* = 3 mice). **k** Pro-inflammatory gene (mRNA) expression in eWAT (*n* = 3 mice). **l** eWAT were lysed for immunoblotting with the indicated antibodies (*n* = 3 mice). **m** Immunoblot analyses of eWAT samples using antibodies against the indicated protein (*n* = 3 mice). **n** Schematic summary of the role of adipocyte TRPM7 in obesity-induced alterations in adipose inflammation and insulin resistance. Statistical data were assessed using two-tailed Student’s *t* test (**b**, **e**–**l**) or one-way ANOVA statistical analysis (**m**) and are presented as mean ± SEM. *p < 0.05. Source data are provided as a Source Data file. kd, relative molecular weight in kilodalton; ns, not significant; veh, vehicle.

In order to examine the effects of pharmacological TRPM7 inhibition in vivo, we administrated oral FTY720 to obese mice for 8 weeks (Fig. 8a). Orally administrated 3 mg/kg FTY720 per day reduced body weight (Fig. 8b, c) and fat weight (Fig. 8d, e). The levels of fasting glucose and serum insulin were significantly reduced after 8 weeks treatment of FTY720 (Fig. 8f, g). FTY720 attenuated serum TG levels but without significance (Fig. 8h). Consistent with these findings, we found that glucose tolerance improved in FTY720-treated mice (Fig. 8i). Pharmacological inhibition of TRPM7 using FTY720 suppressed HFD-induced formation of CLSs, demonstrating a decreased percentage of macrophage in eWAT (Fig. 8j). These changes in FTY720-treated mice were accompanied by downregulation of pro-inflammatory genes (*MCP-1*, *IL6, IL1β*) (Fig. 8k). Western blot analysis showed that activation of IKKβ, NF-κB and IκBα degradation were inhibited by TRPM7 antagonist, whereas insulin-induced phosphorylation of IRS1, IR and AKT were increased (Fig. 8l, m).

## Discussion

In this study, TRPM7 was observed to be an immunometabolic regulator in fat pads that links obesity to inflammation. Adipose-selective ablation of TRPM7 protects mice from exacerbation of inflammation and insulin resistance upon dietary stress. To delineate the underlying mechanisms, we identified that TRPM7-induced rise in intracellular Ca^2+^ as a primary mechanism activated the TAK1-NF-κB cascade, and promoted adipose inflammation and insulin resistance. We demonstrated that TRPM7 mediates TAK1 activation through CaMKII, in cooperation with TRAF6-TAK1 complex stabilization, in a Ca^2+^-triggered manner. Supplementation of TAK1 protein in the eWAT of TRPM7 ATKO mice substantially promoted inflammation while suppressing insulin sensitivity in the adipose tissue. Moreover, FTY720 oral administration in obese mice protected against obesity and insulin resistance. Collectively, our findings suggest a mechanism by which TRPM7-Ca^2+^-TAK1 signaling contributes to adipose function and metabolic homeostasis (Fig. 8n). These findings established adipocyte TRPM7 as a promising pharmacological molecular target for developing therapeutic strategies against obesity and obesity-associated metabolic disorders.

Adipose tissue inflammation contributes to the core mechanisms of several metabolic disorders in obesity, such as insulin resistance, liver steatosis and hyperlipidemia^3^. The inflammation-related effects of TRPM7 on T cell survival, macrophage polarization, vascular calcification, oxidative responses, and cardiac inflammation have been described^16,21,44,45^. TRPM7 is also recognized as a metabolism-associated channel that plays a crucial role in obesity-mediated hypertension^19^. In addition, we provided evidence to show that adipocyte TRPM7 was upregulated primarily in adipocytes, and specific deletion of TRPM7 in adipocytes was sufficient to attenuate HFD-induced obesity, insulin resistance, and inflammation. Moreover, TRPM7-deficient mice exhibited reduced lipid deposition in the liver and BAT, and WAT depot size, which is congruent with clinical correlations between adipocyte size and insulin sensitivity^46^. This indicates that TRPM7 is the major source of pro-inflammatory and pro-obesity signals promoting metabolic disorder development. In obesity, the observed upregulation of TRPM7 in adipose tissue may be in turn elicited by inflammation. Previous studies have shown that upregulation of TRPM7 expression is related to the mRNA levels of TNF-α, which is induced at a very early stage of obesity upon high glucose challenge, contributing to inflammatory responses in diabetes^47^. The promoter region of TRPM7 contains binding sites for NF-κB, STAT3, and other important pro-inflammatory transcription factors^19,48^. Our findings showed that TRPM7 strongly stimulated NF-κB signaling in adipocytes, suggesting that TRPM7 may constitute an essential promoter in a positive feedback loop to fine-tune inflammatory responses in adipocytes, thereby contributing to chronic and low-grade metabolic inflammation in obesity. FTY720, the TRPM7 antagonist and synthetic sphingosine analog, has been clinically used as an immunosuppressant for the treatment of multiple sclerosis^49^. FTY720 has been found to reduce liver steatosis and improve glucose tolerance by downregulating diet-induced fatty acid synthase expression in diet-induced nonalcoholic fatty liver disease^50^. Our finding revealed that FTY720 administration in obese mice could protect against obesity and insulin resistance via changes in adipose tissue inflammation, suggesting that pharmacological inhibition of TRPM7 using FTY720 may also benefit patients with obesity and others with insulin resistance. However, the detailed molecular events resulting in adipose TRPM7 upregulation under overnutrition administration warrant further investigation. Obesity induces a change in circulating or adipose tissue cytokine profile which have direct effects on glucose and lipid metabolism. Recent studies have reported TRPM7 is regulated by cytokine stimulation. IL18 and IL4 upregulate TRPM7 expression and activate the channel activity^24,26^. In contrast, IL6 inhibits TRPM7 inward currents via direct binding with IL6R and the following JAK2/STAT3 pathway activation^51^. Furthermore, TGF-β1-dependent Smad3 cascade promotes TRPM7 transcription to maintain a positive feedback loop in fibrillar collagen production^52^. Serum TNF-α, IL6, IL1β and MCP-1 levels have been identified upregulated in obese mice and modulated by adipocyte TRPM7 knockout in our study, indicating that adipocyte TRPM7 might be regulated by aberrant cytokines with coupled positive-plus-negative feedback circuits in response to obesity.

Obesity has long been known to trigger Ca^2+^ mishandling, linking inflammation to metabolic dysfunction;^53^ however, the identity of the Ca^2+^-permeable channel responsible for initiating the Ca^2+^-signal associated pathway remains unclear. The activity of the store-operated Ca^2+^ entry (SOCE) has been proposed in the setting of obesity and related metabolic diseases^54,55^. SOCE is positively related to cytokine production in adipose tissue macrophages from obese mice^56^, although the loss of SOCE in macrophages does not influence inflammatory signaling^57^. This suggests that SOCE may not be a major determinant in the early phases of Ca^2+^ signaling. Similarly, several TRP Ca^2+^ channels in adipocytes regulate adipocyte biology and insulin sensitivity in obesity^11,56,58,59^. TRPM7 can be modulated by various signals related to inflammation, such as low extracellular pH^20^, oxidative stress^60^, LPS-stimulated TLR4 endocytosis and NF-κB nuclear translocation^25^, and caspase-mediated cleavage in Fas signaling^21^. Our study demonstrated that excessive input of nutrition could activate an outward-rectifying nonselective cation current in adipocytes with an I-V relationship resembling the TRPM7 current, which can be blocked by FTY720. Definitive evidence that TRPM7 acts via Ca^2+^ signal was provided by TRPM7 channel activity inhibition and intracellular Ca^2+^ chelating experiments, which showed that the blockage of TRPM7-dependent-Ca^2+^ influx in adipocytes abolished TNF-α-induced inflammation. TRPM7 has also been reported to regulate cytosolic Ca^2+^ and modulate SOCE by refilling internal Ca^2+^ stores^17,61^. Thus, the processes of releasing intracellular Ca^2+^ or the subsequent SOCE, which may occur concomitantly with TRPM7 channel activity, are not considered salient triggers of the NF-κB cascade and adipose tissue inflammation in obesity. This could be supported by the observation that Ca^2+^ elevations were not completely diminished in TRPM7 knockout adipocytes or under the use of FTY720. Further, our data do not negate the roles of other ion channels in modulating adipocyte functions during obesity. Considering that TRPM7 channel activity is highly sensitive to membrane phosphatidylinositol 4,5-bisphosphonate (PIP2) levels^62^ and phospholipase C (PLC) pathways^63^, TRPM7 activity may be stimulated through these signaling mechanisms in obesity.

To gain insight into the inflammatory processes influenced by TRPM7 in mature adipocytes, we identified the mechanisms involved in Ca^2+^-dependent NF-κB cascade activation. TAK1, a member of the MAPK kinase kinase (MAP3K) family and an essential molecule upstream of IKKs, is central to the activation of the NF-κB pathway^34^. Canonical evidence focused on TAK1 activation suggests that TAK1 serves as an intermediary that mediates inflammatory signaling induced by cytokines (e.g., TNF-α), pathogens (e.g., LPS), hypoxia, or DNA-damage^34,64,65^. Signaling through the TNF-α receptor or TLRs induces TAK1 phosphorylation by recruiting adaptor molecules and the ubiquitin-conjugating system^34^. Recent studies have shown that TAK1 is a lipid senser that orchestrates obesity-linked insulin resistance and inflammatory responses^36,66^. Hyperactivation of TAK1 has been observed upon excessive metabolic stimulation, leading to deteriorated liver inflammation and metabolic profiles^36^. Global or adipose-specific TAK1 deficiency reduced pro-inflammatory immune cells accumulated in WAT, stopped weight gain, improved glucose tolerance, and attenuated hepatic steatosis^66,67^. Restoration of TAK1 kinase activity rescued lipogenic gene expression in hepatocytes, and palmitic acid and oleic acid-induced NF-κB activation was dependent on TAK1 ubiquitination by E3 ubiquitin ligase TRIM8^36,66,67^. Interestingly, transient [Ca^2+^]_i_ and the Ca^2+^ receptor, CaMKII, could determine TAK1 activation and downstream NF-κB signaling activity. Here, we discovered that TAK1 could be activated by TRPM7-induced Ca^2+^ signal, which facilitates NF-κB activation and promotes pro-inflammatory gene expression. Our data indicate that TAK1, downstream of TRPM7, is the pivotal nodal point for obesity-associated inflammation. Moreover, we demonstrated that local injection in the eWAT of TRPM7 ATKO mice with AAV-TAK1 led to striking phenotypes, including strengthened inflammation and mitigated insulin action transduction. These rescue experiments, which aim to reverse the improvement in obesity-linked metabolic disorder by upregulating TAK1, reflect a requirement of TAK1 function in the development of obesity and inflammation states regulated by TRPM7.

Ca^2+^-CaMKII-TAK1, triggered by pro-inflammatory mediators, is involved in the enhanced production of TNF-α and other cytokines in macrophages^33^. Given that CaMKII is indicated as an upstream kinase that directly phosphorylates and activates TAK1^33,34^, and considering our observation that obesity-induced CaMKII activation in adipocytes can be nullified by TRPM7 knockdown or channel activity inhibition, we propose that the activation of CaMKII under a TRPM7-mediated event is the essential Ca^2+^-signaling component of this pathway. However, compared to TRPM7 knockout, only partial inhibition of TAK1-NF-κB cascade activity was mediated by CaMKII inhibitor or CaMKII silencing. In TLR and IL1 pro-inflammatory signaling, TRAF6 is recruited in the MyD88-dependent pathway, is subsequently dimerized, and induces TAK1 ubiquitination via its E3 ligase activity^34^. TRAF6 regulates the NF-κB pathway in several ways, including TRAF6 auto-ubiquitination to initiate TAK1 activation, and catalyzing the ubiquitination of IKKκ/NEMO^43,68^. Similarly, in TNF-α signal transduction, the adaptor TRADD is recruited around the receptor and the ubiquitin-conjugating system, in which the E3 ubiquitin ligase involved varies according to the specific system (e.g., TRAF2, TRAF3, TRAF5, and cIAP1/2 have been reported). These are assembled on the TRADD-receptor scaffold, followed by the K63-linked pUb scaffold and TAK1 activation^34^. In our study, TRPM7 was found to promote TAK1 K63-ubiquitination by the TRAF6 E3 ligase in a Ca^2+^-dependent manner. TRAF6 and CaMKII synergistically regulate TAK1 activation and NF-κB signaling mediated by TRPM7-Ca^2+^ signal. Interestingly, in this study, the EF-hand protein c-Cbl was determined to be a TRPM7-mediated Ca^2+^ signal-triggered molecule regulating TRAF6-TAK1 complex stabilization. C-Cbl, also known as Cbl, functions as an adaptor or E3 ligase and possesses Ca^2+^ binding ability through the EF-hand motif^69^. The c-Cbl interactome can form signal-competent networks that control diverse biological processes, including insulin signaling and inflammatory response^69^. As calcium sensors, EF-hand proteins undergo a conformational change upon binding of calcium, resulting in protein-protein interactions, assembling a proteolytic active site, or stabilizing catalytic complex formation^70^. Structural changes in c-Cbl are required to facilitate adaptor functions or E3 activity. Tyr371 phosphorylation on c-Cbl induces a conformational change in the LHF domain, which converts the RING domain into an enhanced E2-binding module to enhances E3 ligase activity^71^. We propose that during obesity, Ca^2+^ binding-led conformational changes in c-Cbl induce dissociation or altered association of c-Cbl and TRAF6, whereafter TAK1 competes with c-Cbl to bind to TRAF6, thereby facilitating and stabilizing the formation of the TRAF6-TAK1 complex. The hypothesis that the EF-hand protein c-Cbl acts as a Ca^2+^ sensor regulating the assembly of the TRAF6-TAK1 complex might provide a potential explanation, but the detailed mechanisms remain to be further elucidated.

Taken together, our study revealed a previously unappreciated role of adipocyte TRPM7 in regulating adipose inflammation and whole-body metabolism. Adipose-specific ablation of TRPM7 was found to remarkably attenuate adipose inflammation, thereby leading to improved adipose function, insulin sensitivity, and glucose tolerance. Moreover, we demonstrated that TRPM7-dependent-Ca^2+^ signal promotes adipocyte inflammation by TAK1 activation, and CaMKII, in tandem with TRAF6, affects TAK1 activation, leading to NF-κB cascade activation and inflammatory responses. These insights open a line of investigation for a better understanding of adipose inflammation and how TRPM7 modulates glucose homeostasis in obesity.

However, there are several limitations to this study. First, considering that TRPM7-dependent macrophage inflammation has been reported in recent years and that adipose tissue inflammation involves complex mechanisms other than adipocyte dysfunction, such as recruitment of adipose-resident macrophages and T cells, which are regulated by a plethora of immunological and metabolic factors, it remains to be determined whether TRPM7 in inflammatory cells controls adipose tissue inflammation or functions synergistically with adipocytes during obesity development. Second, although we observed body weight loss in adipose-specific TRPM7 knockout mice, our current study did not address the thermogenic functions of TRPM7 in WAT browning or BAT. Third, since only adult male mice were used in this study, the gender difference in TRPM7 function needs to be explored in future. Therefore, further studies on these issues will help us to confirm our current findings.

## Methods

### Ethics statement, animal models, and treatments

All animal experimental procedures were in accordance with Sun Yat-sen University Animal Care and Use Committee-approved protocols, and conformed to the Guide for the Care and Use of Laboratory Animals of National Institute of Health of China. All animal care and procedures were reviewed and approved by Institutional Animal Care and Use Committee of Sun Yat-sen University (SYSU-IACUC-2020-000118). All animals were housed in an air-conditioned room at the room temperature of 25 °C with a 12 h/12 h light/dark circle, and humidity between 40 and 70%, and given ad libitum access to food and water. Only adult male mice were used in our experiments. TRPM7^fl/fl^ (Flox) and Adipoq-Cre mice were purchased from Jackson laboratories (#018784, #028020, Bar Harbor, ME). Adipocyte-specific TRPM7 KO (ATKO) mice were created by breeding TRPM7^fl/fl^ mice to transgenic mice harboring Cre recombinase driven by adiponectin promotor (Adipoq-Cre). The genotypes of mice were determined by PCR using tail DNA. The primer sequences used to determine the genotype are showed in Supplementary Table 1. All the mice have C57BL/6 J background. Animals were anesthetized using pentobarbital sodium (60 mg/kg, intraperitoneal, i.p.) and the tissues were removed for the following experiments set up. Animals were euthanized by cervical dislocation after anaesthetization at the end of the experiments.

Starting at 6-8 weeks of age, Flox and ATKO mice were fed with normal chow diet (CD, 10% kcal fat, D12450J, Research Diets Inc.) or high-fat diet (HFD) consisting of 60% of calories from fat (D12492 Research Diets Inc.) for 16 weeks. For metabolic study, mice were allocated into a calorimetry chamber (Sable Systems, NV, USA) to perform indirect calorimetry study and subject to Inveon multimodality PET/CT system (Inveon Image Research Workplace; Siemens Healthcare, Erlangen, Germany) to perform adipose tissue quantification. Serum insulin, TNF-α, MCP-1, IL-6 and IL-1β levels were measured with ELISA kit (RayBiotech, Norcross, GA, USA) according to the manufacture’s protocol.

For administration of AAV8-TAK1 vector, AAV8-Adipoq-Cre vector or AAV8-Vec to epididymal adipose tissue, mice aged 6–8 weeks were anesthetized with pentobarbital sodium (60 mg/kg) intraperitoneally and the laparotomy was performed. AAV8-TAK1 vector, AAV8-Adipoq-Cre vector and AAV8-Vec were obtained from Weizhen biosciences (Shandong, China). Each epididymal fat pad was given 8 injections of 5 ul (1 × 10^13^ viral genome copies) of AAV solution.

### Primary mature adipocytes isolation

Epididymal adipose tissues from male mice were removed and weighted, rinsed in phosphate-buffered saline (PBS) for 3 times and minced in PBS. Tissue suspensions were digested in DMEM containing 1 mM CaCl_2_, 1 mM MgCl_2_, 8 mg/ml collagenase D and 2.4 units/ml Dispase II (Sigma-Aldrich) for 30 min and filtered through a 220 μm filter and centrifugated at 200 *g* for 3 min. The top floating fractions which contained mature adipocytes were collected. Adipocytes were washed with DMEM/F12 medium supplemented with 10% FBS, 100 IU penicillin and 100 mg/L streptomycin by repetitive pelleting. For mature adipocytes, the final adipocyte suspension was then used for the subsequent experiments.

### Stromal vascular fraction isolation, differentiation and FACS analysis

Epididymal fat pads were prepared and digested as described above. To isolate SVF, the pellet was collected and washed with DMEM/F12 medium supplemented with 10% FBS, 100 IU penicillin and 100 mg/L streptomycin by repetitive pelleting. SVFs were used for subsequent culture and differentiation and fluorescence-activated cell sorting analysis (FACS). Primary mouse SVFs or 3T3-L1 cells (purchased from National Collection of Authenticated Cell Cultures, China, catalog number: SCSP-5038) were cultured to confluence in DMEM/F12 medium containing 10% FBS (SVFs) or 10% newborn calf serum (3T3-L1 cells), 100 IU penicillin and 100 mg/L streptomycin and then induced to differentiation with 5 μg/mL insulin, 1 μM dexamethasone, 0.5 mM 3-isobutyl-methylxanthine, and 1 μM rosiglitazone. For FACS, the SVFs were incubated with RBC lysis buffer for 5 min followed by centrifugation at 300 *g* for 5 min and resuspension in FACS buffer. The SVFs were incubated with Fcγ receptor block for 20 min at 4 °C before staining with fluorescence labeled primary antibodies (FITC anti-F4/80, Biolegend, 123107, 0.2 μg per 10^6^ cells in 100 μl volume; PE/Cy5 anti CD11b, Biolegend, 101210, 0.2 μg per 10^6^ cells in 100 μl volume) for 20 min at 4 °C. Flow cytometry was performed using CytoFLEX flow cytometer (Beckman Coulter Ltd., Brea, CA, USA) and fluorescence signals were analyzed using FlowJo 10.4 software.

### Adipose tissue and liver histology analysis

Samples of adipose tissue and livers were fixed in 4% formaldehyde for 24 h and cut into small pieces and processed for paraffin embedding. 5-μm sections were stained with hematoxylin and eosin and the adipocyte size was evaluated by using Image J 1.52q software. For immunohistochemical staining of F4/80, adipose sections were incubated with primary antibody against F4/80 overnight at 4 °C and then stained with biotinylated secondary antibody for 1 h at room temperature followed by visualization with 2.2-diaminobenzidine tetrachloride. Liver samples were frozen in OCT embedding medium and 10-μm sections were used to stain with Oil Red O to observe lipid accumulation in liver. All images were acquired using optical microscopy (Olympus, Tokyo, Japan).

### Metabolic studies

For glucose tolerance tests (GTTs), mice received an intraperitoneal (i.p.) injection with glucose (1 g/kg) after fasted overnight for 16 h and blood glucose concentrations were drawn to measure at 0, 30, 60, 90 and 120 min after glucose injection. For insulin tolerance tests (ITTs), after 6 h of fasting, insulin (0.75 U/kg) was i.p. injected and blood glucose was measured at 0, 15, 30, 45, and 60 min after insulin injection. We performed acute insulin challenge experiments on anesthetized mice after 6 h fasting. The samples of fat, liver, and muscle were snap-frozen for protein analysis at 20 min after injection with insulin (0.75 U/kg; i.p.).

### Currents recording

TRPM7 currents were recorded in whole-cell configuration using Axopatch 200B patch clamp amplifier (Axon Instrument, USA) as described previously^14,72,73^. Patch pipettes with resistances between 3 and 6 MΩ were made from borosilicate glass by using Sutter P-97 horizontal puller (Sutter instrument, Novato, CA, USA). The standard external solution contained: 145 mM NaCl, 2 mM CaCl_2_, 5 mM KCl, 10 mM Glucose, 10 mM HEPES, pH 7.3 (adjusted with NaOH). The standard pipette solution contained: 145 mM Cs-methanesulfonate, 8 mM NaCl, 10 mM Cs-EGTA, 10 mM HEPES, pH 7.3 (adjusted with CsOH). Cells were held at a potential of 0 mV and the voltage ramps from −100 mV to +100 mV at the duration of 50 ms and intervals of 2 s were applied for whole-cell recording. Membrane currents were analyzed using pCLAMP 10.0 software (Axon Instruments).

### Ca^2+^ imaging

Freshly isolate adipocytes from eWAT were incubated in a standard bath solution containing 140 mM NaCl, 5 mM KCl, 1 MgCl_2_, 10 mM Glucose, 10 mM HEPES (pH 7.4) and loaded with 5 μM Fluo-4 AM for 45 min in the dark at 37 °C. Adipocytes were excited at 488 nm and emissions were measured at 530 nm. Fluorescence was detected using a Zeiss LSM880 microscope (Carl Zeiss, Oberkochen, Germany). For ratiometric Ca^2+^ imaging, adipocytes were bathed in Ca^2+^, Mg^2+^-free solution (145 mM NaCl, 5 mM KCl, 10 mM Glucose, 10 mM HEPES, pH 7.3) for 4 min and then CaCl_2_ to a final concentration of 10 mM was added to determine Ca^2+^ influx.

### Transfection

The siRNA duplexes against mouse CaMKII, TRAF6 and TRPM7 gene along with scramble RNA were synthesized by RiboBio (Guangzhou, China) (Supplementary Table 2). siRNA strands or plasmid vectors (1 μg) were used to transiently transfect HEK293T cells (purchased from National Collection of Authenticated Cell Cultures, China, catalog number: SCSP-502) or adipocytes with Lipofectamine 3000 (Life Technologies, Carlsbad, CA, USA), according to the manufacturer’s instruction. 48 h later, cells were harvested for the following experiments.

### RNA isolation, quantitative PCR and RNA-Seq

Total RNA was isolated from tissues or cells using RNA purification kit (Qiagen). Complementary DNA (cDNA) was synthesized using the QuantiTect Reverse Transcription Kit (Qiagen, Hilden, Germany). Quantitative PCR (qPCR) reaction was set up using SYBR Green PCR Master Mix (Invitrogen, Carlsbad, CA) and the amplification curves were monitored on the LightCycler 480 (Roche, Basel, Switzerland). The qPCR primers used are listed in Supplementary Table 3 and were synthesized by Sangon (Shanghai, China). All measurements were carried out in triplicate with β-actin as an internal standard and 2-△△Ct method was used to calculate the expression of target genes. RNA-seq analysis was performed by Majorbio Bio-pharm Technology Co., Ltd (Shanghai, China).

### Western blot analysis, Immunoprecipitation and ubiquitination assay

Western blot analysis and immunoprecipitation were carried out as described previously^73–75^. Total proteins were prepared from the tissues and cells with RIPA buffer containing protease and phosphatase inhibitors. The lysates were subjected to western blotting and the protein was separated with SDS-PAGE and transferred to PVDF transfer membranes (Millipore, Bedford, MA, USA). Corresponding antibodies were used to detect proteins. The protein bands were measured by ChemiDoc^TM^ Touch Imaging System (Bio-Rad, Richmond, CA, USA). All experiments were repeated at least three times and amount of phosphorylated protein was quantified by Image J and normalized to amount of total protein.

For immunoprecipitation and ubiquitination, HEK293T and adipocytes were homogenized in lysis buffer. 1 mg of cell lysate protein was immunoprecipitated with indicated antibodies at 4 °C overnight. Immunoprecipitations were pulled down with Protein A/G Agarose followed by western blot analysis. For ubiquitination assays, cells were lysed with lysis buffer plus 2 mM N-Ethylmaleimide (NEM) and denudated by heating for 10 min, and centrifuged at 14,000 g for 15 min at 4 °C. The subsequent steps for the in vivo ubiquitination assay were carried out as described above in methods of immunoprecipitation and western blotting experiments.

The following antibodies were used: anti-TRPM7 (Sigma-Aldrich, AB15562), WB 1:500; anti-phospho-IRS-1 (Ser307) (Cell Signaling Technology, 2381), WB 1:1000; anti-IRS-1 (Cell Signaling Technology, 2382), WB 1:1000; anti-phospho-Insulin Receptor β (Tyr1345) (Cell Signaling Technology, 3026), WB 1:1000; anti-Insulin Receptor β (Cell Signaling Technology, 3025), WB 1:1000; anti-phospho-Akt (Ser473) (Cell Signaling Technology, 4060), WB 1:1000; anti-Akt (Cell Signaling Technology, 9272), WB 1:1000; anti-phospho-NFκB-p65 (Ser536) (Cell Signaling Technology, 3033), WB 1:1000; anti-NFκB-p65 (Cell Signaling Technology, 8242),WB 1:1000; anti-phospho-IKKβ (Ser180) (Cell Signaling Technology, 2694), WB 1:1000; anti-IKKβ (Cell Signaling Technology, 8943),WB 1:1000; anti-IκBα (Cell Signaling Technology, 9242), WB 1:1000; anti-phospho-TAK1 (Ser412) (Cell Signaling Technology, 9339), WB 1:1000; anti-TAK1 (Cell Signaling Technology, 5206), WB 1:1000, IP 1:50; anti-phospho-CaMKII (Thr286) (Cell Signaling Technology, 12716) WB 1:1000; anti-CaMKII (Cell Signaling Technology, 50049) WB 1:1000; anti-Ub (Cell Signaling Technology, 3936), WB 1:1000; anti-Flag (Sigma, F1804), WB 1:1000, IP 10 μl for 1 mg protein; anti-HA (Sigma, H3663), WB 1:1000; anti-TRAF6 (Santa Cruz, sc-8409), WB 1:1000, IP 4 μg for 1 mg protein; anti-c-Cbl (Santa Cruz, sc-1651), WB:1:1000; anti-GAPHD (Proteintech, 60004-1-Ig), WB 1:2000; anti-α-tubulin (Proteintech, 11224-1-AP), WB 1:2000; anti-rabbit (Cell Signaling Technology, 7074), WB 1:2000; anti-mouse (Cell Signaling Technology, 7076), WB 1:2000.

### Statistical analysis

All the replicate experiments are biological replicants which were repeated at least three times. Raw data were applied directly in statistical analysis. All data are expressed as mean ± SEM. All analyses were performed by using SPSS 25 and GraphPad Prism 8 software. Comparison of two groups was determined by Student’s *t* test for independent samples and multiple groups by one-way ANOVA. All statistical tests were two-tailed and the level for statistical significance was 0.05.

### Reporting summary

Further information on research design is available in the Nature Portfolio Reporting Summary linked to this article.

## Supplementary information

Supplementary Information

Reporting Summary

## Data Availability

The experimental data generated during this study are available from the Lead Contact (Min Gao, gaom9@mail.sysu.edu.cn) upon request without restrictions. The RNA-seq data generated in this study have been deposited in the NCBI’s Sequence Read Archive (SRA) database with the accession code PRJNA884894 and the Genome Sequence Archive (GSA) database under accession number CRA008343. Source data are provided with this paper.

## Source data

Source Data
