# Supplementary material for: Adipose-specific deletion of the cation channel TRPM7 inhibits TAK1 kinase-dependent inflammation and obesity in male mice
Source: Nat Commun. 2023 Jan 30;14:491. doi: 10.1038/s41467-023-36154-3 (PMC9887063; doi:10.1038/s41467-023-36154-3)
Supplement: Supplementary file 1 — Supplementary Information [file 41467_2023_36154_MOESM1_ESM.pdf]

Adipose-specific deletion of the cation channel TRPM7 inhibits TAK1 kinase-dependent inflammation and obesity in male mice

Weiting Zhong[1,#], Mingming Ma[2,#], Jingwen Xie[1], Chengcui Huang[1], Xiaoyan Li[1,*], Min Gao[1,#,*]

[1] Department of Pharmacy, the Sixth Affiliated Hospital, Sun Yat-sen University, Guangzhou 510655, China

[2] Department of Pharmacology, Cardiac and Cerebral Vascular Research Center, Zhongshan School of Medicine, Sun Yat-sen University, Guangzhou 510080, China

[#] These authors contributed equally: Min Gao, Weiting Zhong, Mingming Ma

[*] Correspondence: lixyan5@mail.sysu.edu.cn; gaom9@mail.sysu.edu.cn (Lead Contact)

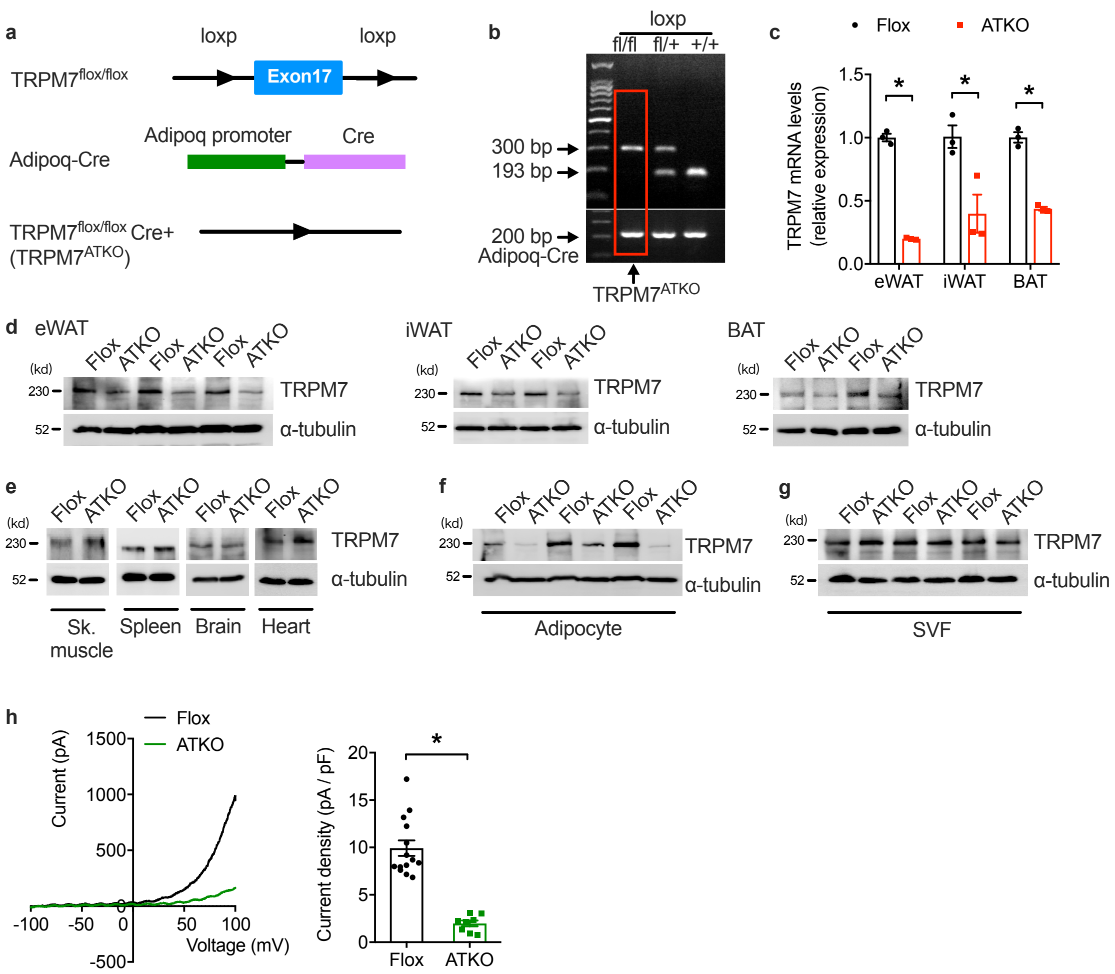

Supplementary Fig. 1. Generation of adipose-specific TRPM7-knockout mice. a. Schematic diagram of transgenic mice used to generate ATKO ($TRPM7^{ATKO}$) mice. Adipocyte-specific knockout strategy where triangles designate LoxP sites flanking exon 17 of TRPM7. **b.** Mice were identified by PCR amplified from mouse genomic DNA using primers specific for Loxp-1, Loxp-2 and Adipoq-Cre respectively. **c.** qPCR analysis of TRPM7 mRNA expression in adipose tissue from Flox and ATKO mice (n=3 mice). **d-g.** Western blot analysis of TRPM7 protein level in adipose tissues (**d,** n=3 mice), skeletal muscle, spleen, brain and heart (**e,** n=3 mice), isolated adipocytes (**f,** n=3 mice) and stromal vascular fraction (SVF) (**g,** n=3 mice) from Flox and ATKO mice. **h.** A representative I-V relationship of whole-cell I_{TRPM7} in freshly isolated adipocytes from Flox and ATKO mice (n=14 cells for Flox, n=8 cells for ATKO, from three mice each group). The statistics of TRPM7 current densities were quantified in bar chart are shown. Statistical data were assessed using two-sided Student's t test (Supplementary Fig. 1c and 1h) and are presented as mean ± SEM. *p<0.05. Source data are provided as a Source Data file. kd, relative molecular weight in kilodalton.

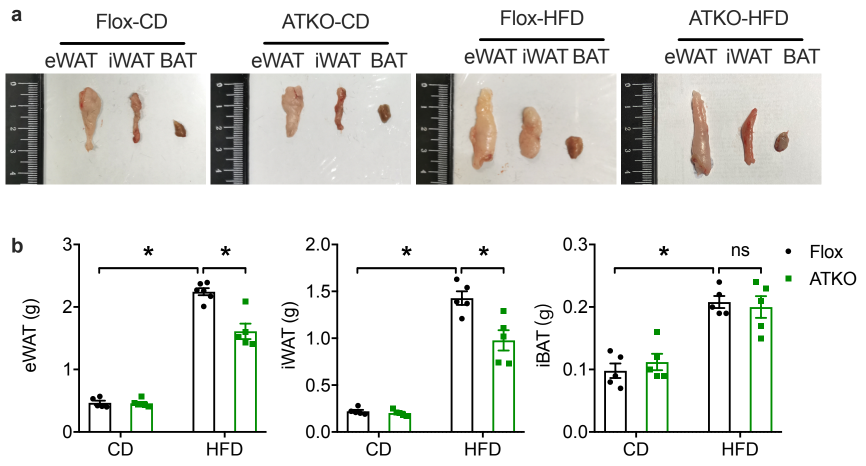

Supplementary Fig. 2. TRPM7 deficiency decreases white adipose tissue weight. a. Representative photographs of fat pads (eWAT, iWAT and BAT). **b.** The weight of eWAT (n=5 mice for Flox-CD and ATKO-HFD, n=6 mice for ATKO-CD and Flox-HFD), iWAT (n=5 mice) and BAT (n=5 mice). Statistical data in **b** were assessed using one-way ANOVA statistical analysis (Supplementary Fig. 2b) and are presented as mean ± SEM. *$p<0.05$. Source data are provided as a Source Data file.

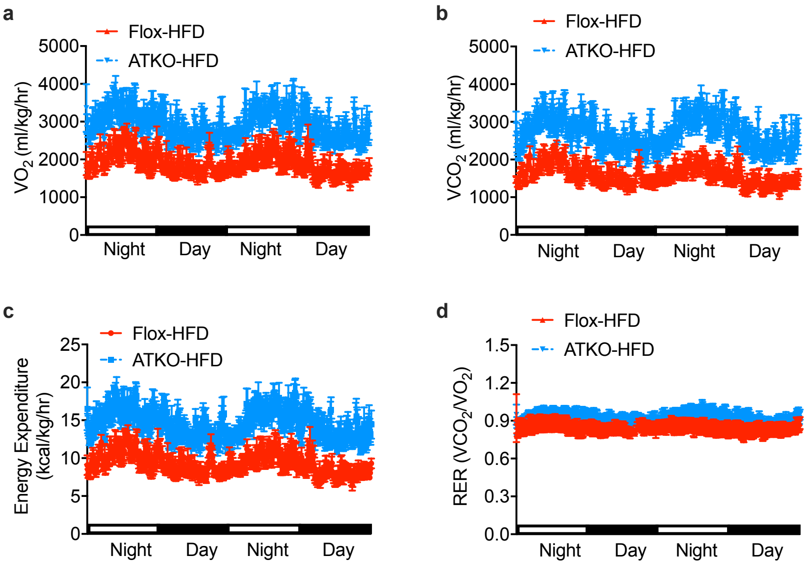

Supplementary Fig. 3. TRPM7 ablation increases basic metabolic activity. Related to Fig. 1. TRPM7 knockout protects against diet-induced obesity by increasing energy expenditure. Whole-body oxygen consumption (VO_2) (**a**), carbon dioxide production (VCO_2) (**b**), respiratory exchange ratio (VCO_2/VO_2) (**c**) and energy expenditure rate (**d**) monitored continuously over a 24-h period (n=6 mice). The average VO_2, VCO_2 VCO_2/VO_2 and energy expenditure rate during 12-h light/12-h dark periods are showed in Fig. 1. Statistical data are presented as mean ± SEM. Source data are provided as a Source Data file.

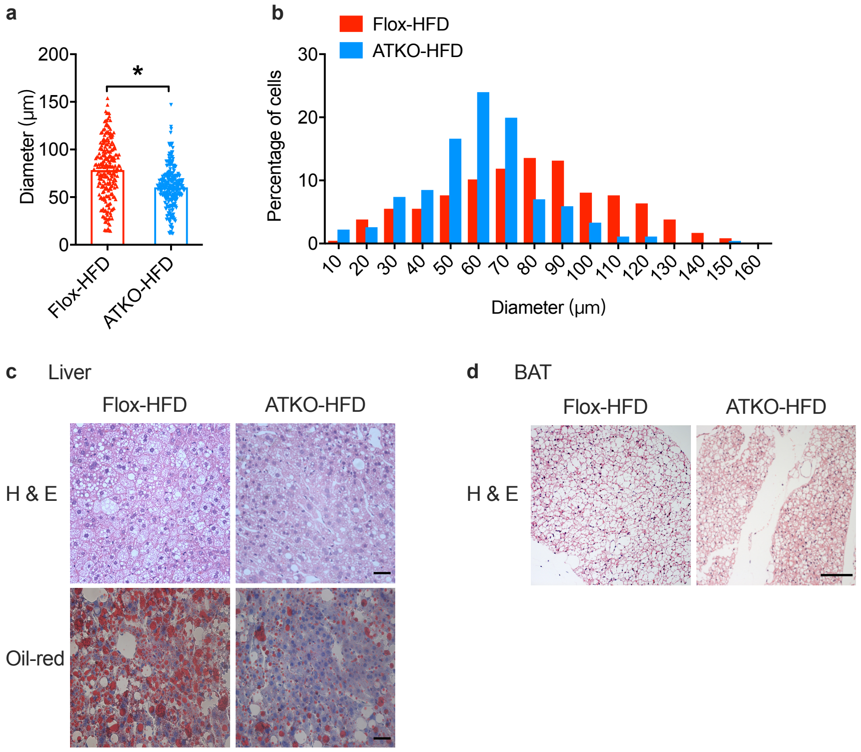

Supplementary Fig. 4. TRPM7 ATKO mice display a reduced adiposity and are resistant to diet-induced hepatic steatosis. a-b. Quantification of adipocyte diameter and distribution of Flox and ATKO mice fed with CD or HFD (n=236 cells from 4 mice for Flox-HFD, n=271 cells from 6 mice for ATKO-HFD). **c-d.** Representative H&E images of liver, liver oil red O staining (**c**), and H&E images of BAT (**d**) from the indicated groups (scale bar, 100 μm) (n=3 mice). Statistical data in **a** were assessed using two-sided Student's t test (Supplementary Fig. 4a) and are presented as mean ± SEM. *$p<0.05$. Source data are provided as a Source Data file.

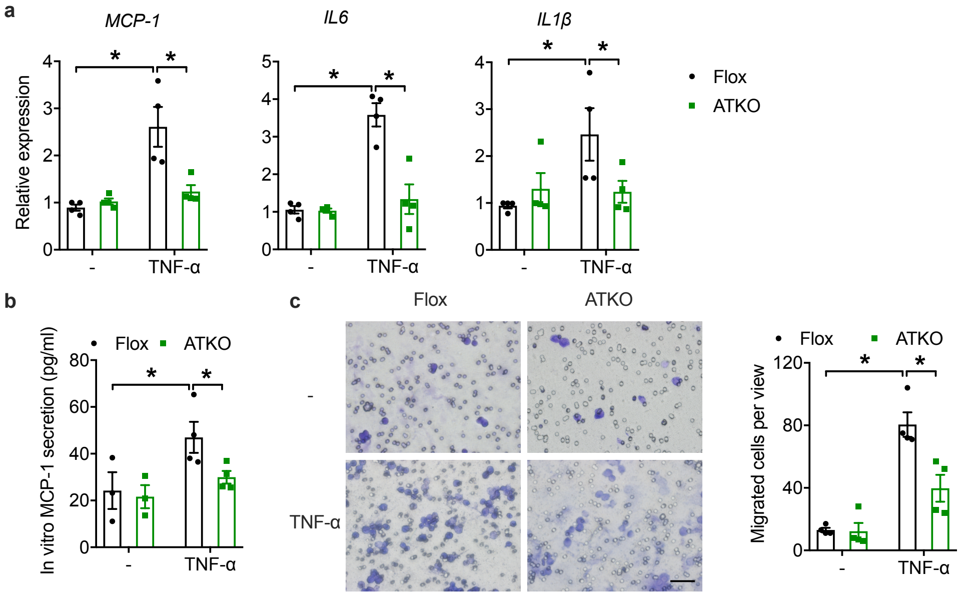

Supplementary Fig. 5. TRPM7 knockdown attenuates adipocyte inflammation. Related to Fig. 3. **a.** The indicated gene expression gene expression of *MCP-1*, *IL6*, *IL1β* in primary adipocytes (n=4 biologically independent experiments). **b.** Protein secretion of MCP-1 in adipocytes treated with or without TNF-α (n=3 biologically independent experiments treated without TNF-α, n=4 biologically independent experiments treated with TNF-α). **c.** Effect of conditional medium from Flox and ATKO primary adipocytes treated with or without TNF-α on macrophage chemotaxis (scale bar, 50 μm) (n=4 biologically independent experiments). Statistical data were assessed using one-way ANOVA statistical analysis (Supplementary Fig. 5a-5c) and are presented as mean ± SEM. *p<0.05. Source data are provided as a Source Data file.

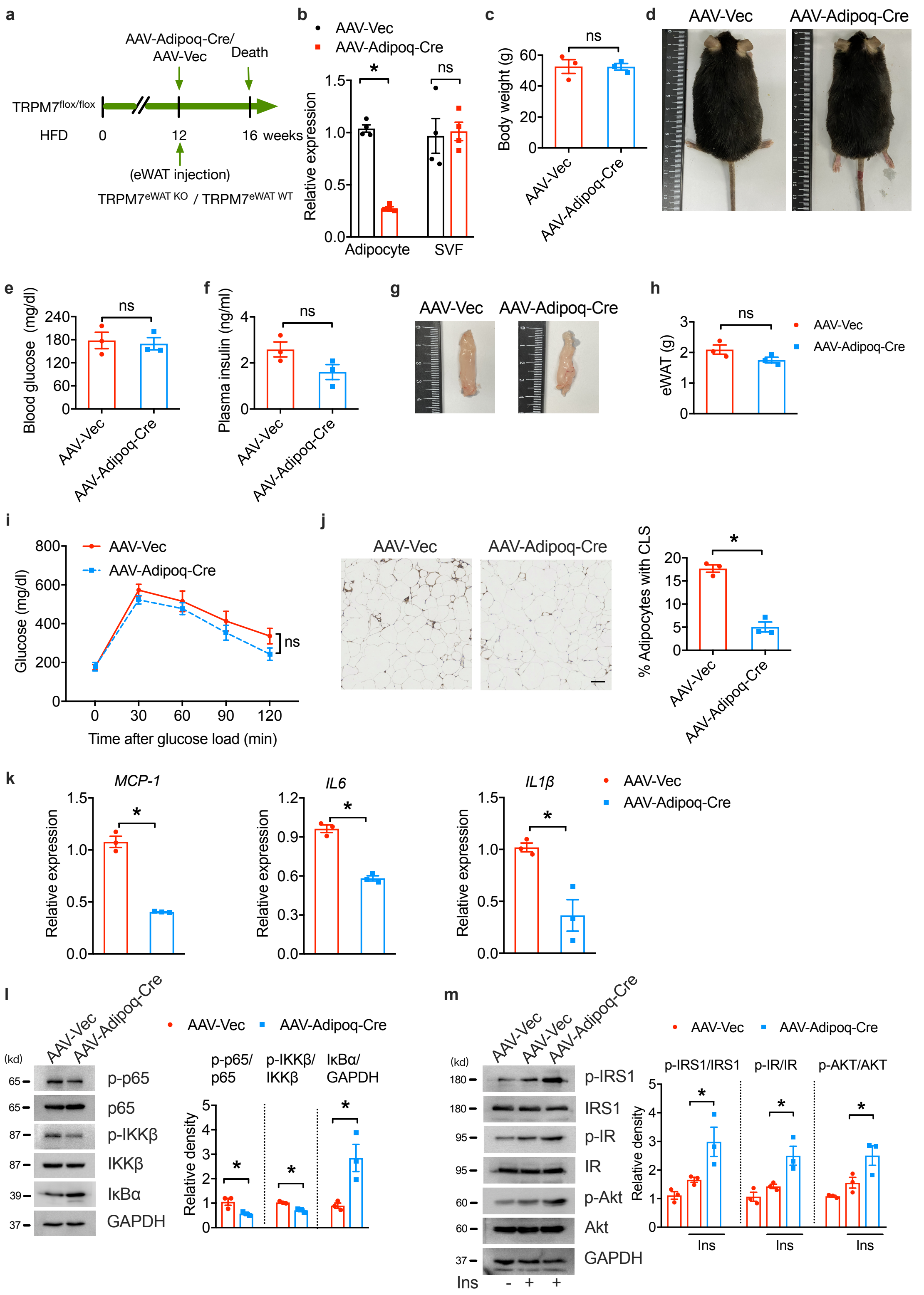

Supplementary Fig. 6. TRPM7 deficiency mitigates adipose inflammation independent of body weight loss. a. Schematic representation of TRPM7$^{eWAT\ KO}$ mice by AAV-Cre administration. **b.** TRPM7 mRNA levels in isolated adipocytes and SVF from eWAT of TRPM7$^{eWAT\ WT}$ (WT) and TRPM7$^{eWAT\ KO}$ mice after induction (n=4 mice). **c.** Body weight of HFD WT and TRPM7$^{eWAT\ KO}$ (n=3 mice). **d.** Representative photographs of WT and TRPM7$^{eWAT\ KO}$ mice fed with HFD (n=3 mice). **e-f.** Blood glucose and insulin levels (n=3 mice). **g-h.** Weights of eWAT between WT and

TRPM7$^{eWAT\ KO}$ mice (n=3 mice). **i.** TRPM7 knockout in eWAT slightly ameliorates glucose intolerance with no significance (n=3 mice). **j.** Macrophages were stained by using an antibody to F4/80. ATMs (adipose tissue macrophages) were quantitated by measurement of F4/80 staining of tissue sections using Image J software (scale bar, 100 μm) (n=3 mice). **k.** The relative expression of the proinflammation genes were measured by qPCR assays of mRNA (n=3 mice). **l.** Phosphorylation of IKKβ and NF-κB p65 and IκBα expression in eWAT (n=3 mice). **m.** The WT and TRPM7$^{eWAT\ KO}$ mice were fasted overnight, treated by i.p. injection with 0.75 U/kg insulin, and examined by immunoblot analysis of eWAT by probing with antibodies to phospho-IRS1, phospho-IR, phospho-AKT, IRS1, IR and AKT (n=3 mice). Statistical data were assessed using two-sided Student's t test (Supplementary Fig. 6b, 6c, 6e, 6f, 6h-6l) or one-way ANOVA statistical analysis (Supplementary Fig. 6m) and are presented as mean ± SEM. *$p<0.05$. Source data are provided as a Source Data file. kd, relative molecular weight in kilodalton; ns, not significant; vec, vector.

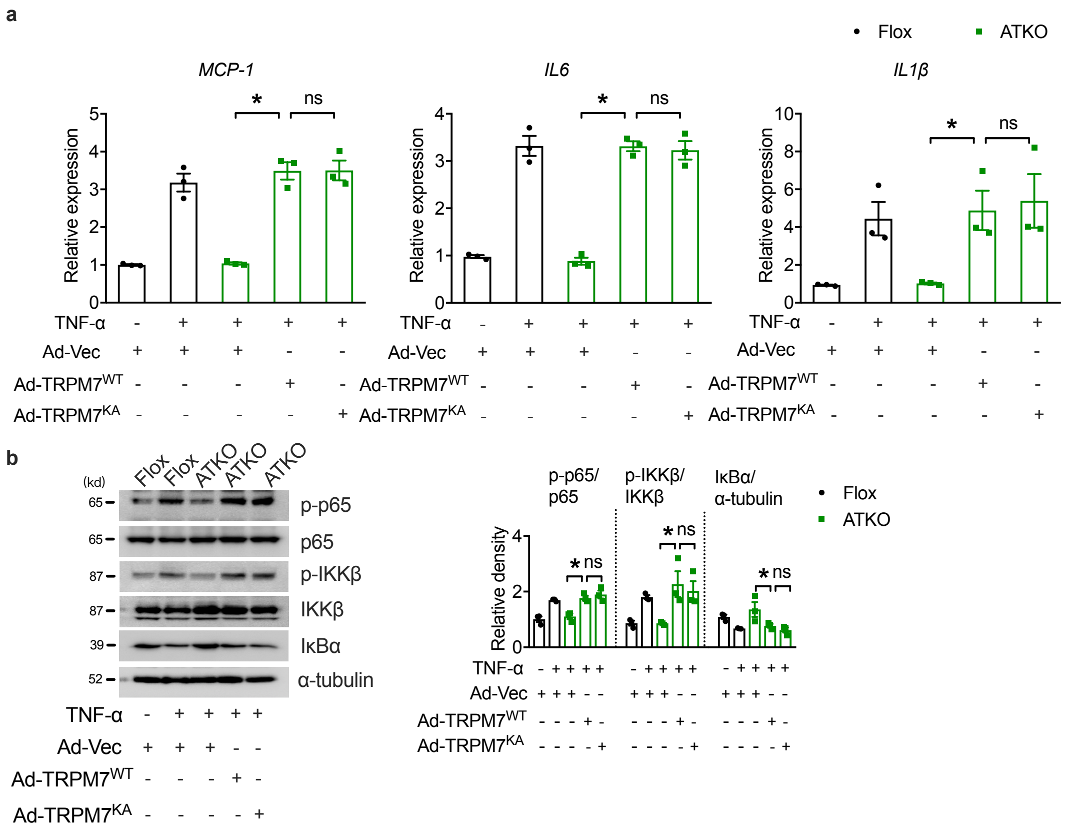

Supplementary Fig. 7. The kinase activity of TRPM7 does not take part in adipocyte inflammation. a. Cultured primary adipocytes were transfected with TRPM7 adenovirus (TRPM7 wild type, Ad-TRPM7^{WT}) or TRPM7 kinase dead mutant adenovirus (K1646A, Ad-TRPM7^{KA}), and their control adenovirus (Ad-Vec) respectively. Adipocytes were treated with TNF-α (40 ng/ml) for 12 h and gene expression of *MCP-1*, *IL6*, *IL1β* was measured using qPCR (n=3 biologically independent experiments). **b.** Adipocytes were lysed for immunoblotting with the indicated antibodies (n=3 biologically independent experiments). Statistical data were assessed using one-way ANOVA statistical analysis (Supplementary Fig. 7a-7b) and are presented as mean ± SEM. *p<0.05. Source data are provided as a Source Data file. kd, relative molecular weight in kilodalton.

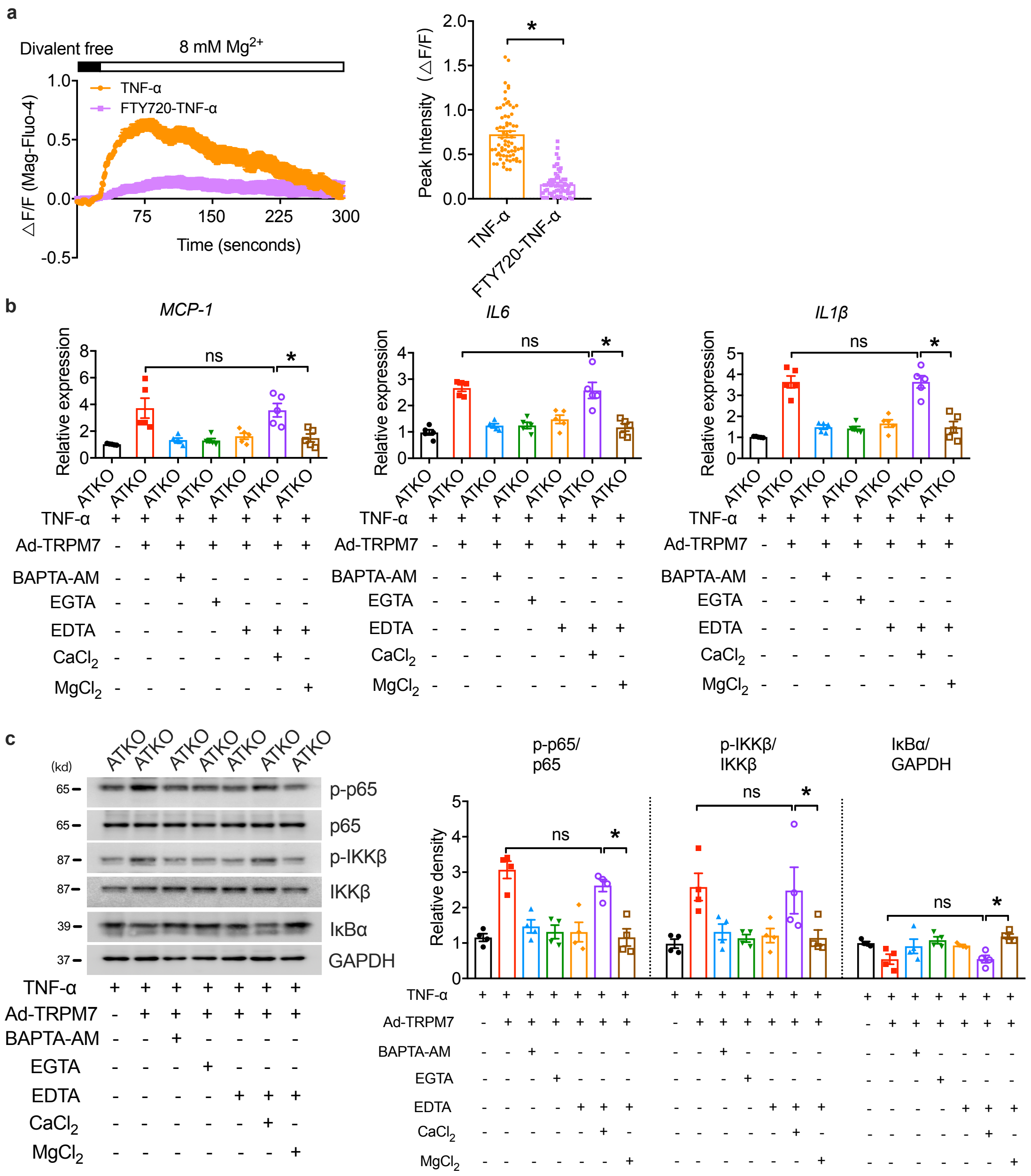

Supplementary Fig. 8. TRPM7-dependent Mg^{2+} influx does not account for adipocyte inflammation. a. Time-course changes in intracellular Mg^{2+} ($[Mg^{2+}]_i$) quantified by measuring the peak fluorescence intensity. Adipocytes were incubated in divalent-free solution (DVF, Ca^{2+}, and Mg^{2+}-free) supplemented with 4 µM Mag-Fluo-4 (ThermoFisher Scientific) in the dark for 30 min. Real-time images were captured using a Zeiss LSM880 microscope (Carl Zeiss, Oberkochen, Germany). Adipocytes were bathed in DVF medium with addition of 8 mM $MgCl_2$ to measure Mg^{2+} influx (n=69 cells treated with TNF-α, n=61 cells treated with FTY720+TNF-α, from three independent experiments). **b.** Adipocytes were incubated in DMEM/F12 medium supplemented with EDTA (5 mM), EGTA (5 mM), BAPTA-AM (10 µM), excess Ca^{2+} (15 mM) over EDTA (5 mM), or excess Mg^{2+} (15 mM) over EDTA (5 mM), respectively, and treated with TNF-α (40 ng/ml; 12 h). qPCR analysis revealed that increasing Ca^{2+} (excess Ca^{2+} over EDTA) but not Mg^{2+} (excess Mg^{2+} over EDTA) in adipocytes reproduced the TNF-α-induced inflammatory genes expression found in control adipocytes (n=5 biologically independent experiments). **c.** Western blots analysis

of the indicated proteins in adipocytes stimulation with TNF-α (60 ng/ml) for 15 min (n=4 biologically independent experiments). Statistical data were assessed using two-sided Student's t test (Supplementary Fig. 8a) or one-way ANOVA statistical analysis (Supplementary Fig. 8b-8c) and are presented as mean ± SEM. *$p<0.05$. Source data are provided as a Source Data file. kd, relative molecular weight in kilodalton; ns, not significant.

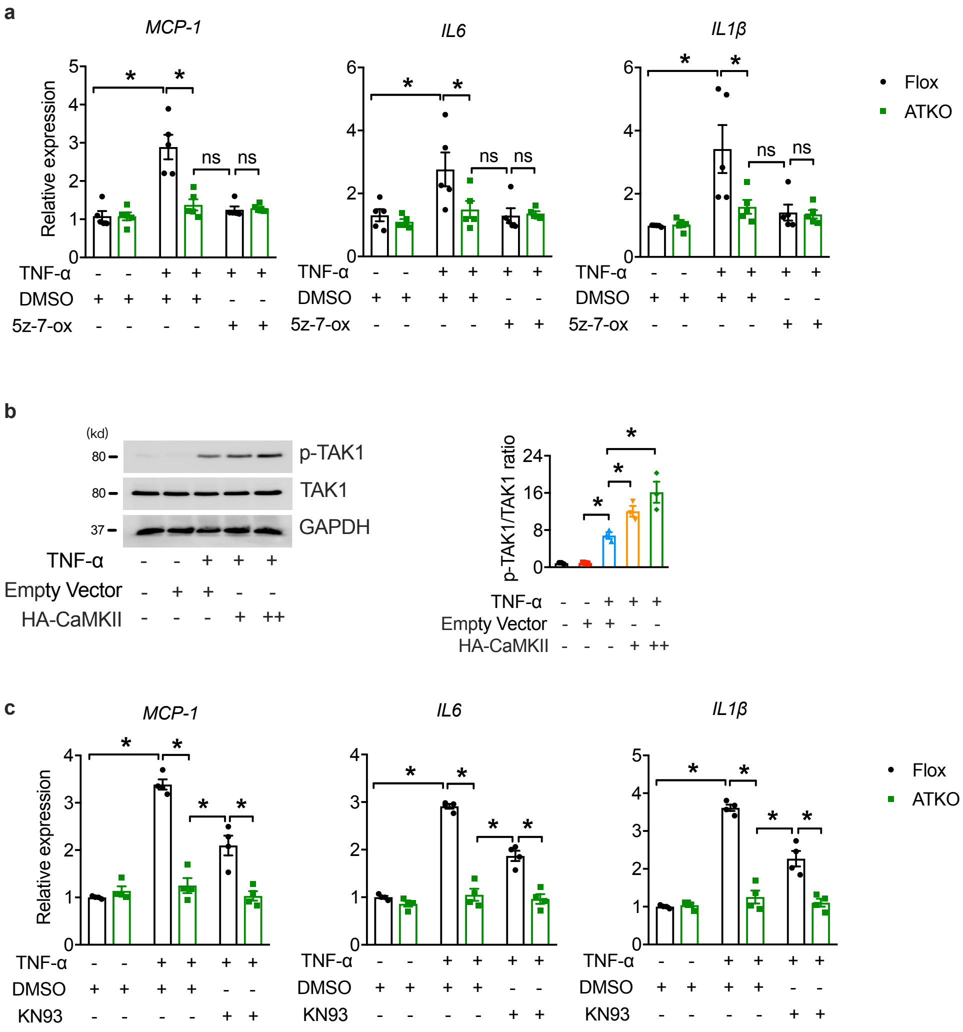

Supplementary Fig. 9. CaMKII-TAK1 cascade participates in TRPM7-dependent inflammation. Related to Fig. 5. **a.** Gene expression of *MCP-1*, *IL6*, *IL1β* in differentiated adipocytes incubated with DMSO or TAK1 inhibitor 5z-7-ox (100 nM) for 30 min, then stimulated with TNF-α (40 ng/ml; 12 h) or not (n=5 biologically independent experiments). **b.** Immunoblot analysis of 3T3-L1 adipocytes transiently overexpressing HA-CaMKII for 24 h, followed with or without 60 ng/ml TNF-α treatment for 15 min (n=3 biologically independent experiments). **c.** qPCR analysis of relative *MCP-1*, *IL6*, *IL1β* mRNA levels in differentiated adipocytes pretreated with CaMKII inhibitor KN93 (10 μM; 30 min) before stimulating with TNF-α (40 ng/ml; 12 h) (n=4 biologically independent experiments). Statistical data were assessed using one-way ANOVA statistical analysis (Supplementary Fig. 9a-9c) and are presented as mean ± SEM. *p<0.05. Source data are provided as a Source Data file. kd, relative molecular weight in kilodalton; ns, not significant.

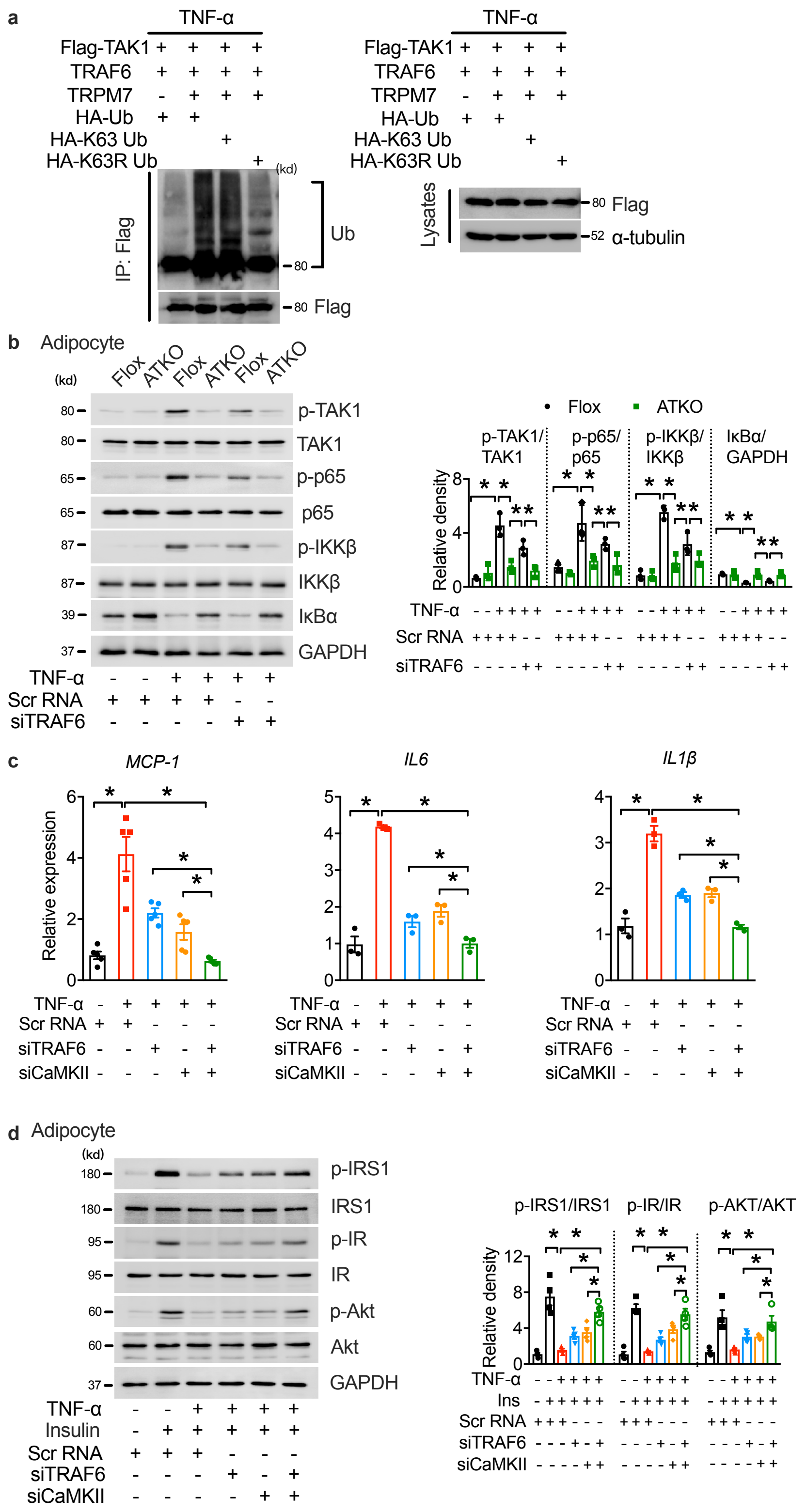

Supplementary Fig. 10. Both TRAF6 and CaMKII together mediates TRPM7-dependent adipocyte inflammation. Related to Fig. 6. **a.** Ubiquitination levels of TAK1 after overexpression of TRAF6 and Flag-TAK1 and in response to TNF-α treatment (60 ng/ml; 30 min) in HEK293T cells cotransfected with TRPM7 and the indicated HA-tagged K63 or K63R ubiquitin. K63R indicates ubiquitin at K63 was mutated (n=3 biologically independent experiments). **b.** Phosphorylated and total TAK1, p65, IKKβ, IκBα in primary adipocytes of Flox and ATKO mice

challenged with TNF-α (60 ng/ml; 15 min) (n=3 biologically independent experiments). **c.** The levels of indicated mRNA were analyzed by qPCR. Differentiated 3T3-L1 adipocytes were transfected with TRAF6 siRNA, CaMKII siRNA or scramble siRNA as indicated for 24 hours and then treated with TNF-α (n=5 biologically independent experiments for *MCP-1*, n=3 biologically independent experiments for *IL6* and *IL1β*). **d.** Insulin (100 nM; 15 min)-stimulated activation of insulin signaling was examined by western blotting in 3T3-L1 adipocytes transfected with TRAF6 siRNA, CaMKII siRNA or scramble siRNA and treated with TNF-α (60 ng/ml; 15 min) (n=4 biologically independent experiments). The data were assessed using one-way ANOVA (Supplementary Fig. 10b-10d) and are presented as mean ± SEM. *p<0.05. Source data are provided as a Source Data file. kd, relative molecular weight in kilodalton.

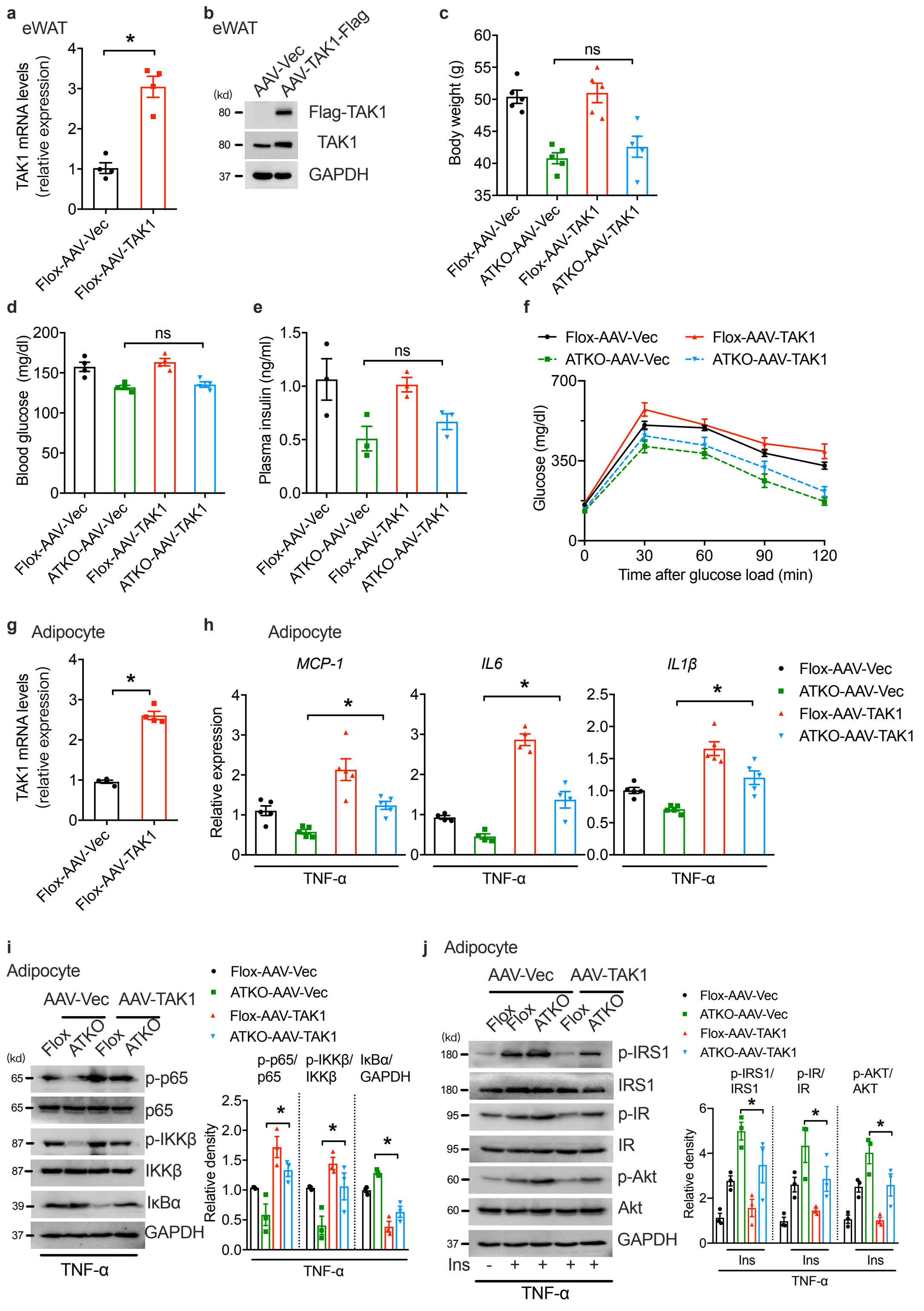

Supplementary Fig. 11. Adipose TAK1 overexpression augments adipose inflammation in TRPM7 knockout obese mice. Related to Fig. 7. **a-b.** The mRNA (**a**) and protein level (**b**) of TAK1 in eWAT of HFD-fed mice injected with Ad-Flag or Ad-TAK1-Flag (n=4 mice). **c-e.** Body weight (**c,** n=5 mice), fasting blood glucose (**d,** n=4 mice) and insulin (**e,** n=3 mice) levels of HFD-fed Flox and ATKO mice injected with AAV-Vec or AAV-TAK1. **f.** Glucose tolerance test on the indicated groups of mice (n=4 mice). **g.** The mRNA of TAK1 in isolated adipocytes from eWAT of HFD-fed

mice injected with AAV-Vec or AAV-TAK1 (n=4 mice). **h.** The levels of indicated mRNA in cultured primary adipocytes were analyzed by qPCR (n=5 mice for *MCP-1* and *IL1β*, n=4 mice for *IL6*). **i.** The expression of proteins in NF-κB signaling cascade in the adipocytes isolated from the indicated groups. The isolated adipocytes were incubated with TNF-α (60 ng/ml; 15 min) (n=3 mice). **j.** Insulin (100 nM; 15 min)-stimulated activation of insulin signaling was examined by western blotting in isolated adipocytes from the indicated groups. The adipocytes were incubated with TNF-α (60 ng/ml; 15 min) prior to insulin stimulation (n=3 mice). Statistical data were assessed using two-sided Student's t test (Supplementary Fig. 11a and 11g) or one-way ANOVA statistical analysis (Supplementary Fig. 11c-11f, 11h-11j) and are presented as mean ± SEM. *$p<0.05$. Source data are provided as a Source Data file. kd, relative molecular weight in kilodalton; ns, not significant.

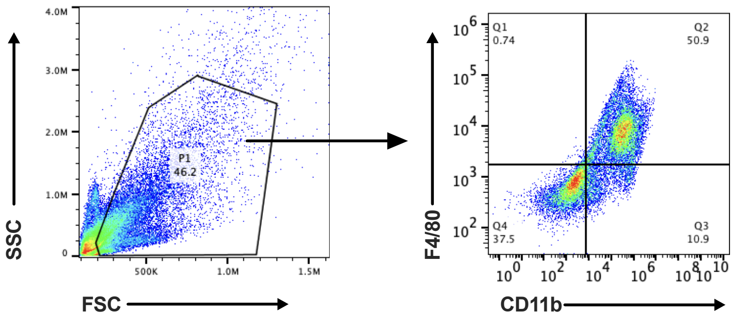

Supplementary Fig. 12. Gating strategy of flow cytometry for analysis of adipose tissue macrophage number.

Supplementary Table 1. Sequences of the primers for TRPM7 LoxP and Adipoq-Cre mice genotyping.

Primer name	Forward primer sequence (5’to 3’)	Reverse primer sequence (5’to 3’)
LoxP	TTTCTCCAATTAGCCCTGTAG A	CTTGCCATTTTACCCAAATC
Adipoq-Cre	ACGGACAGAAGCATTTTCCA	GGATGTGCCATGTGAGTCTG

Supplementary Table 2. Target sequences of siRNAs.

Target Name	Target Sequence
CaMKII	CACCACCATTGAGGACGAA
TRAF6	CATTAAGGATGATACATTA
TRPM7	CCTGATGAGGTTGTCACAG

Supplementary Table 3. Gene primers used for qPCR.

Genes	Primer sequences
mCcl2 F	TTAAAAACCTGGATCGGAACCAA
mCcl2 R	GCATTAGCTTCAGATTTACGGGT
mTnfa F	TATGGCTCAGGGTCCAACTC
mTnfa R	CTCCCTTTGCAGAACTCAGG
mIL6 F	AGTTGCCTTCTTGGGACTGA
mIL6 R	CAGAATTGCCATTGCACAAC
mIL1b F	GCCCATCCTCTGTGACTCAT
mIL1b R	AGGCCACAGGTATTTTGTCG
b-actin F	ACTGTGCCCATCTACGAGG
b-actin R	CAGGCAGCTCATAGCTCTT
mTAK1 F	AGGTTGTCGGAAGAGGAGCT
mTAK1 R	CTCCACAATGAAAGCCTTCC
mF4/80 (Adgre1) F	ACCACAATACCTACATGCACC
mF4/80 (Adgre1) R	AAGCAGGCGAGGAAAAGATAG
mTRPM7 F	TTTGGTGTTCCCAGAAAAGC
mTRPM7 R	ACCAAGTTCCAGGACCACAG
