# Supplementary material for: Adipose-specific deletion of the cation channel TRPM7 inhibits TAK1 kinase-dependent inflammation and obesity in male mice
Source: Nat Commun. 2023 Jan 30;14:491. doi: 10.1038/s41467-023-36154-3 (PMC9887063; doi:10.1038/s41467-023-36154-3)
Supplement: Supplementary file 2 — Reporting Summary [file 41467_2023_36154_MOESM2_ESM.pdf]

nature portfolio

Corresponding author(s): Min Gao

Last updated by author(s): Dec 6, 2022

Reporting Summary

Nature Portfolio wishes to improve the reproducibility of the work that we publish. This form provides structure for consistency and transparency in reporting. For further information on Nature Portfolio policies, see our Editorial Policies and the Editorial Policy Checklist.

Statistics

For all statistical analyses, confirm that the following items are present in the figure legend, table legend, main text, or Methods section.

n/a	Confirmed	
☐	☒	The exact sample size (*n*) for each experimental group/condition, given as a discrete number and unit of measurement
☐	☒	A statement on whether measurements were taken from distinct samples or whether the same sample was measured repeatedly
☐	☒	The statistical test(s) used AND whether they are one- or two-sided<br>*Only common tests should be described solely by name; describe more complex techniques in the Methods section.*
☒	☐	A description of all covariates tested
☐	☒	A description of any assumptions or corrections, such as tests of normality and adjustment for multiple comparisons
☐	☒	A full description of the statistical parameters including central tendency (e.g. means) or other basic estimates (e.g. regression coefficient) AND variation (e.g. standard deviation) or associated estimates of uncertainty (e.g. confidence intervals)
☐	☒	For null hypothesis testing, the test statistic (e.g. *F*, *t*, *r*) with confidence intervals, effect sizes, degrees of freedom and *P* value noted<br>*Give P values as exact values whenever suitable.*
☒	☐	For Bayesian analysis, information on the choice of priors and Markov chain Monte Carlo settings
☒	☐	For hierarchical and complex designs, identification of the appropriate level for tests and full reporting of outcomes
☒	☐	Estimates of effect sizes (e.g. Cohen's *d*, Pearson's *r*), indicating how they were calculated

Our web collection on statistics for biologists contains articles on many of the points above.

Software and code

Policy information about availability of computer code

Data collection	The images of H&E, IHC were obtained using optical microscopy (Olympus, Tokyo, Japan). Ca2+ imaging was conducted using a Zeiss LSM880 microscope (Carl Zeiss, Oberkochen, Germany). Ct values for qPCR data were calculated by LightCycler 480 PCR system (Roche, Swizerland). Western blot band intensity was analyzed using ChemiDocTM Touch Imaging System (Bio-Rad, Richmond, CA, USA). Flow cytometry was performed using CytoFLEX flow cytometer (Beckman Coulter Ltd., Brea, CA, USA). TRPM7 currents were recorded in whole cell configuration using Axopatch 200B patch clamp amplifier (Axon Instrument, USA). Membrane currents were analyzed using pCLAMP 10.0 software (Axon Instruments). For metabolic study, mice were allocated into a calorimetry chamber (Sable Systems, NV, USA) to perform indirect calorimetry study and subject to Inveon multimodality PET/CT system (Inveon Image Research Workplace; Siemens Healthcare, Erlangen, Germany) to perform adipose tissue quantification.
Data analysis	We utilized GraphPad Prism 8, FlowJo 10.4, SPSS 25, and Image J 1.52q to perform data analysis.

For manuscripts utilizing custom algorithms or software that are central to the research but not yet described in published literature, software must be made available to editors and reviewers. We strongly encourage code deposition in a community repository (e.g. GitHub). See the Nature Portfolio guidelines for submitting code & software for further information.

Data

Policy information about availability of data

All manuscripts must include a data availability statement. This statement should provide the following information, where applicable:

- Accession codes, unique identifiers, or web links for publicly available datasets
- A description of any restrictions on data availability
- For clinical datasets or third party data, please ensure that the statement adheres to our policy

The authors declare that all the source data are provided as a Source Data file. Raw data of RNA-seq has been deposited in the NCBI's Sequence Read Archive (SRA) data base with the accession code PRJNA884894 (https://www.ncbi.nlm.nih.gov/sra/?term=PRJNA884894) and the Genome Sequence Archive (GSA) database under accession number CRA008343 (https://bigd.big.ac.cn/gsa/browse/CRA008343).

Human research participants

Policy information about studies involving human research participants and Sex and Gender in Research.

Reporting on sex and gender	N/A
Population characteristics	N/A
Recruitment	N/A
Ethics oversight	N/A

Note that full information on the approval of the study protocol must also be provided in the manuscript.

Field-specific reporting

Please select the one below that is the best fit for your research. If you are not sure, read the appropriate sections before making your selection.

☒ Life sciences ☐ Behavioural & social sciences ☐ Ecological, evolutionary & environmental sciences

For a reference copy of the document with all sections, see nature.com/documents/nr-reporting-summary-flat.pdf

Life sciences study design

All studies must disclose on these points even when the disclosure is negative.

Sample size	Sample size determination is based on the previous experience to obtain significance and reproducibility (Gao, Hypertension, 2022). The sample size following common standards employing three or more biological replicates. All sample sizes are listed in each figure legend.
Data exclusions	No data exclusions.
Replication	All experiments were performed at least three times in an independent manner. All the attempts at replication were successful.
Randomization	Samples were randomly allocated in this study.
Blinding	Investigators were blinded to group allocation during the data collection and analysis.

Reporting for specific materials, systems and methods

We require information from authors about some types of materials, experimental systems and methods used in many studies. Here, indicate whether each material, system or method listed is relevant to your study. If you are not sure if a list item applies to your research, read the appropriate section before selecting a response.

Materials & experimental systems

n/a	Involved in the study
☐	☒ Antibodies
☐	☒ Eukaryotic cell lines
☒	☐ Palaeontology and archaeology
☐	☒ Animals and other organisms
☒	☐ Clinical data
☒	☐ Dual use research of concern

Methods

n/a	Involved in the study
☒	☐ ChIP-seq
☐	☒ Flow cytometry
☒	☐ MRI-based neuroimaging

Antibodies

Antibodies used

anti-TRPM7 Sigma-Aldrich AB15562 WB 1:500
anti-phospho-IRS-1 (Ser307) Cell Signaling Technology 2381 WB 1:1000
anti-IRS-1 Cell Signaling Technology 2382 WB 1:1000
anti-phospho-Insulin Receptor β (Tyr1345) Cell Signaling Technology 3026 WB 1:1000
anti-Insulin Receptor β Cell Signaling Technology 3025 WB 1:1000
anti-phospho-Akt (Ser473) Cell Signaling Technology 4060 WB 1:1000
anti-Akt Cell Signaling Technology 9272 WB 1:1000
anti-phospho-NFκB-p65 (Ser536) Cell Signaling Technology 3033 WB 1:1000
anti-NFκB-p65 Cell Signaling Technology 8242 WB 1:1000
anti-phospho-IKKβ (Ser180) Cell Signaling Technology 2694 WB 1:1000
anti-IKKβ Cell Signaling Technology 8943 WB 1:1000
anti-IκBα Cell Signaling Technology 9242 WB 1:1000
anti-phospho-TAK1 (Ser412) Cell Signaling Technology 9339 WB 1:1000
anti-TAK1 Cell Signaling Technology 5206 WB 1:1000 IP 1:50
anti-phospho-CaMKII (Thr286) Cell Signaling Technology 12716 WB 1:1000
anti-CaMKII Cell Signaling Technology 50049 WB 1:1000
anti-Ub Cell Signaling Technology 3936 WB 1:1000
anti-Flag Sigma F1804 WB 1:1000 IP 10 μl for 1 mg protein
anti-HA Sigma H3663 WB 1:1000
anti-TRAF6 Santa Cruz sc-8409 WB 1:1000 IP 4 μg for 1 mg protein
anti-c-Cbl Santa Cruz sc-1651 WB:1:1000
anti-GAPHD Proteintech 60004-1-Ig WB 1:2000
anti-α-tubulin Proteintech 11224-1-AP WB 1:2000
anti-rabbit Cell Signaling Technology 7074 WB 1:2000
anti-mouse Cell Signaling Technology 7076 WB 1:2000
FITC anti-F4/80 Biolegend 123107 0.2 μg per 10^6 cells in 100 μl volume
PE/Cy5 anti CD11b Biolegend 101210 0.2 μg per 10^6 cells in 100 μl volume

Validation

The antibodies used in this study are commercially available and validated by their respective manufacturers.
anti-TRPM7: https://www.sigmaaldrich.cn/CN/zh/product/mm/ab15562

anti-phospho-IRS-1 (Ser307): https://www.cellsignal.cn/products/primary-antibodies/phospho-irs-1-ser307-antibody/2381?site-search-type=Products&N=4294956287&Ntt=2381&fromPage=plp&_requestid=3095978

anti-IRS-1: https://www.cellsignal.cn/products/primary-antibodies/irs-1-antibody/2382?site-search-type=Products&N=4294956287&Ntt=2382&fromPage=plp&_requestid=3096156

anti-phospho-Insulin Receptor β (Tyr1345): https://www.cellsignal.cn/products/primary-antibodies/phospho-insulin-receptor-b-tyr1345-14a4-rabbit-mab/3026?site-search-type=Products&N=4294956287&Ntt=3026&fromPage=plp&_requestid=3096500

anti-Insulin Receptor β: https://www.cellsignal.cn/products/primary-antibodies/insulin-receptor-b-4b8-rabbit-mab/3025?site-search-type=Products&N=4294956287&Ntt=3025&fromPage=plp&_requestid=3096593

anti-phospho-Akt (Ser473): https://www.cellsignal.cn/products/primary-antibodies/phospho-akt-ser473-d9e-xp-rabbit-mab/4060?site-search-type=Products&N=4294956287&Ntt=4060&fromPage=plp&_requestid=3096743

anti-Akt: https://www.cellsignal.cn/products/primary-antibodies/akt-antibody/9272?site-search-type=Products&N=4294956287&Ntt=9272&fromPage=plp&_requestid=3096948

anti-phospho-NFκB-p65 (Ser536): https://www.cellsignal.cn/products/primary-antibodies/phospho-nf-kb-p65-ser536-93h1-rabbit-mab/3033?site-search-type=Products&N=4294956287&Ntt=3033&fromPage=plp&_requestid=3097105

anti-NFκB-p65: https://www.cellsignal.cn/products/primary-antibodies/nf-kb-p65-d14e12-xp-rabbit-mab/8242?site-search-type=Products&N=4294956287&Ntt=8242&fromPage=plp&_requestid=3097248

anti-phospho-IKKβ (Ser180): https://www.cellsignal.cn/products/primary-antibodies/phospho-ikka-b-ser176-180-antibody-ii/2694?site-search-type=Products&N=4294956287&Ntt=2694&fromPage=plp&_requestid=3097358

anti-IKKβ: https://www.cellsignal.cn/products/primary-antibodies/ikkb-d30c6-rabbit-mab/8943?site-search-type=Products&N=4294956287&Ntt=8943&fromPage=plp&_requestid=3097475

anti-IκBα: https://www.cellsignal.cn/products/primary-antibodies/ikba-antibody/9242?site-search-type=Products&N=4294956287&Ntt=9242&fromPage=plp&_requestid=3098806

anti-phospho-TAK1 (Ser412): https://www.cellsignal.cn/products/primary-antibodies/phospho-tak1-ser412-antibody/9339?site-search-type=Products&N=4294956287&Ntt=9339&fromPage=plp&_requestid=3098904

anti-TAK1: https://www.cellsignal.cn/products/primary-antibodies/tak1-d94d7-rabbit-mab/5206?site-search-type=Products&N=4294956287&Ntt=5206&fromPage=plp&_requestid=3093170

anti-phospho-CaMKII (Thr286): https://www.cellsignal.cn/products/primary-antibodies/phospho-camkii-thr286-d21e4-rabbit-mab/12716?site-search-type=Products&N=4294956287&Ntt=12716&fromPage=plp&_requestid=3090563

anti-CaMKII: https://www.cellsignal.cn/products/primary-antibodies/camkii-a-6g9-mouse-mab/50049?site-search-type=Products&N=4294956287&Ntt=50049&fromPage=plp&_requestid=3093433

anti-Ub: https://www.cellsignal.cn/products/primary-antibodies/ubiquitin-p4d1-mouse-mab/3936?site-search-type=Products&N=4294956287&Ntt=3936&fromPage=plp&_requestid=3093595

anti-Flag: https://www.sigmaaldrich.cn/CN/zh/product/sigma/f1804

anti-HA: https://www.sigmaaldrich.cn/CN/zh/product/sigma/h3663

anti-TRAF6: https://www.scbt.com/p/traf6-antibody-d-10?requestFrom=search

anti-c-Cbl: https://www.scbt.com/p/cbl-antibody-a-9?requestFrom=search

anti-GAPHD: https://www.ptgcn.com/products/GAPDH-Antibody-60004-1-Ig.htm

anti-α-tubulin: https://www.ptgcn.com/products/TUBA1B-Antibody-11224-1-AP.htm

anti-rabbit: https://www.cellsignal.cn/browse/?N=4294956287&Ntk=Products&Ntt=7074&site-search-type=Products

anti-mouse: https://www.cellsignal.cn/products/secondary-antibodies/anti-mouse-igg-hrp-linked-antibody/7076?site-search-type=Products&N=4294956287&Ntt=7076&fromPage=plp&_requestid=6674387

FITC anti-F4/80: https://www.biolegend.com/en-us/products/fitc-anti-mouse-f4-80-antibody-4067

PE/Cy5 anti CD11b: https://www.biolegend.com/en-us/products/pe-cyanine5-anti-mouse-human-cd11b-antibody-350

Eukaryotic cell lines

Policy information about cell lines and Sex and Gender in Research

Cell line source(s)	3T3-L1 cells and HEK293T cells were obtained from National Collection of Authenticated Cell Cultures.
Authentication	Cells were routinely authenticated by morphological examination using microscopy.
Mycoplasma contamination	All cell lines were confirmed negative for mycoplasma contamination.
Commonly misidentified lines (See ICLAC register)	None.

Animals and other research organisms

Policy information about studies involving animals; ARRIVE guidelines recommended for reporting animal research, and Sex and Gender in Research

Laboratory animals	Mouse strains used in this study:<br>TRPM7 flox/flox, C57BL/6J Background, purchased from Jackson laboratories (Bar Harbor, ME)<br>Adipoq-Cre, C57BL/6J Backgound, purchased from Jackson laboratories (Bar Harbor, ME)<br>wild-type C57BL/6J, purchased from Cyagen (Shenzhen, China)<br>All mice with age 6-8 weeks were used in this study.
Wild animals	The study did not involve any wild animals.
Reporting on sex	Only adult male mice were used in our experiments. Female mice are resistant to HFD-induced weight gain and insulin resistance. Epididymal white adipose tissue (eWAT) from male mice was used as a representative visceral adipose tissue which is considered as a link between obesity and the related metabolic inflammation.
Field-collected samples	The study did not use field-collected samples.

Ethics oversight

All animal experimental procedures were in accordance with Sun Yat-Sen University Animal Care and Use Committee-approved protocols, and conformed to the Guide for the Care and Use of Laboratory Animals of National Institute of Health of China. All animal care and procedures were reviewed and approved by Institutional Animal Care and Use Committee of Sun Yat-Sen University (SYSU-IACUC-2020-000118).

Note that full information on the approval of the study protocol must also be provided in the manuscript.

Flow Cytometry

Plots

Confirm that:

☒ The axis labels state the marker and fluorochrome used (e.g. CD4-FITC).

☒ The axis scales are clearly visible. Include numbers along axes only for bottom left plot of group (a 'group' is an analysis of identical markers).

☒ All plots are contour plots with outliers or pseudocolor plots.

☒ A numerical value for number of cells or percentage (with statistics) is provided.

Methodology

Sample preparation

Epididymal adipose tissue from male mice were removed and weighted, rinsed in phosphate-buffered saline (PBS) for 3 times and minced in PBS. Tissue suspensions were digested in DMEM containing 1 mM CaCl2, 1 mM MgCl2, 8 mg/ml collagenase D and 2.4 units/ml Dispase II (Sigma-Aldrich) for 30 min and filtered through a 220 µm filter and centrifugated at 200 g for 3 min. The pellet was collected and washed with DMEM/F12 medium supplemented with 10% FBS, 100 IU penicillin and 100 mg/L streptomycin by repetitive pelleting. SVFs were used for fluorescence-activated cell sorting analysis (FACS). The SVFs were incubated with RBC lysis buffer for 5 min followed by centrifugation at 300 g for 5 min and resuspension in FACS buffer. The SVFs were incubated with Fcγ receptor block for 20 min at 4 ? before staining with fluorescence labeled primary antibodies for 20 min at 4 ?. Fluorescence signals were analyzed using FlowJo software.

Instrument

Cells were analyzed on Beckman CytoFLEX.

Software

The data were analyzed with FlowJo 10.4.

Cell population abundance

Cell sorting was not performed in this study. Macrophages were sorted directly from dissociated adipose tissue on F4/80 +CD11b+.

Gating strategy

FSC and SSC were used to gate the targeted cell population. Cells were analyzed by cell specific gating using primary antibodies, followed by fluorescence-labeled secondary antibodies. CD11b and F4/80 double positive cells were gated as macrophages based on the isotype controls.

☒ Tick this box to confirm that a figure exemplifying the gating strategy is provided in the Supplementary Information.
